# Sedimentary record from Patagonia, southern Chile supports cosmic-impact triggering of biomass burning, climate change, and megafaunal extinctions at 12.8 ka

**DOI:** 10.1038/s41598-018-38089-y

**Published:** 2019-03-13

**Authors:** Mario Pino, Ana M. Abarzúa, Giselle Astorga, Alejandra Martel-Cea, Nathalie Cossio-Montecinos, R. Ximena Navarro, Maria Paz Lira, Rafael Labarca, Malcolm A. LeCompte, Victor Adedeji, Christopher R. Moore, Ted E. Bunch, Charles Mooney, Wendy S. Wolbach, Allen West, James P. Kennett

**Affiliations:** 1 Instituto de Ciencias de la Tierra, Universidad Austral de Chile, Valdivia, Chile; 2 Transdiciplinary Center for Quaternary Research (TAQUACH), Universidad Austral de Chile, Valdivia, Chile; 3 Departamento de Antropología, Universidad Católica de Temuco, Temuco, Chile; 4 Laboratorio Pilauco, Universidad Austral de Chile, Valdivia, Chile; 5 Elizabeth City State University, Center of Excellence in Remote Sensing Education and Research, Elizabeth City, NC 27909 USA; 6 Department of Natural Sciences, Elizabeth City State University, Elizabeth City, NC 27909 USA; 7 Savannah River Archaeological Research Program, South Carolina Institute of Archaeology and Anthropology, University of South Carolina, Columbia, South Carolina 29208 USA; 8 Geology Program, School of Earth Science and Environmental Sustainability, Northern Arizona University, Flagstaff, AZ 86011 USA; 9 Analytical Instrumentation Facility, North Carolina State University, Raleigh, NC 27695 USA; 10 Department of Chemistry, DePaul University, Chicago, IL 60614 USA; 11 Comet Research Group, Prescott, AZ 86301 USA; 12 Department of Earth Science and Marine Science Institute, University of California, Santa Barbara, CA 93106 USA

## Abstract

The Younger Dryas (YD) impact hypothesis posits that fragments of a large, disintegrating asteroid/comet struck North America, South America, Europe, and western Asia ~12,800 years ago. Multiple airbursts/impacts produced the YD boundary layer (YDB), depositing peak concentrations of platinum, high-temperature spherules, meltglass, and nanodiamonds, forming an isochronous datum at >50 sites across ~50 million km² of Earth’s surface. This proposed event triggered extensive biomass burning, brief impact winter, YD climate change, and contributed to extinctions of late Pleistocene megafauna. In the most extensive investigation south of the equator, we report on a ~12,800-year-old sequence at Pilauco, Chile (~40°S), that exhibits peak YD boundary concentrations of platinum, gold, high-temperature iron- and chromium-rich spherules, and native iron particles rarely found in nature. A major peak in charcoal abundance marks an intense biomass-burning episode, synchronous with dramatic changes in vegetation, including a high-disturbance regime, seasonality in precipitation, and warmer conditions. This is anti-phased with northern-hemispheric cooling at the YD onset, whose rapidity suggests atmospheric linkage. The sudden disappearance of megafaunal remains and dung fungi in the YDB layer at Pilauco correlates with megafaunal extinctions across the Americas. The Pilauco record appears consistent with YDB impact evidence found at sites on four continents.

## Introduction

The YDB impact hypothesis posits that the hemisphere-wide debris field of a large, fragmenting asteroid/comet struck Earth and caused brief “impact winter” conditions^1–3^. In turn, this triggered major shifts in continental drainage patterns, dramatically changed oceanic circulation, and caused abrupt global climate changes (temperature and precipitation) marked by the Younger Dryas (YD) climatic episode in the Northern Hemisphere^4^. The airburst/impacts are proposed to have caused significant environmental changes, including widespread biomass burning, anomalously abrupt shifts in plant and animal distributions, human cultural changes and population declines, and broad extinctions within the iconic late Pleistocene megafauna^1^. Studies of the Younger Dryas boundary (YDB) layer report peak abundances of a diverse suite of proposed cosmic impact-related proxies at more than 50 sites, located mostly within the Northern Hemisphere, but with two previous sites in the Southern Hemisphere (Venezuela^5,6^ and Antarctica^7^). These include peak abundances of high-temperature high-iron (Fe) spherules, glassy silica-rich spherules, high-temperature meltglass, nanodiamonds, platinum (Pt), iridium (Ir), osmium (Os), and/or other impact-related proxies (for references, cf. Supplementary Table A2 in Wolbach *et al*.^3^). The YDB layer is also often marked by abundance peaks in biomass-burning proxies, including charcoal, aciniform carbon/soot, carbon spherules, glasslike carbon and/or combustion aerosols^1–3,8–12^. This entire suite of proxies at the YD onset is argued to represent one of the largest known biomass-burning peaks of the late Quaternary^2,3^.

First proposed in 2007^1^, the YDB impact hypothesis is still controversial a decade later because a number of independent studies have raised questions about the proposed YDB impact event. (1) The authors of several studies^13–15^ have argued that dating accuracy and precision are insufficient to determine whether YDB proxies are coeval across the many sites. (2) Five of 13 independent studies were unable to confirm the presence of peaks in YDB magnetic spherules (see Supplementary Information, “Previously reported evidence of YDB magnetic spherules” for details). (3) Three studies^13–15^ noted that sediment sampling at YDB sites is typically discontinuous, except at and around the YDB layer, where sampling spanned at most a few thousand years, making it difficult to know whether the “impact assemblage” of indicators is unique to the YDB (Supplementary Table S1). The same concern applies to this study in which we sampled continuously at high resolution for ~300 years across the YDB layer and sporadically before and after, totalling ~17,000 years. (4) In one study^13^, it was argued that the proposed YDB impact mechanism of a fragmented comet is so rare as to be statistically implausible. (5) Several investigators^15–18^ proposed that the rate and timing of megafaunal extinctions were not simultaneous but varied across the continents, with a large percentage of them occurring prior to the YDB. (6) Another study^19^ concluded that a major peak in wildfire activity preceded the YD onset by several hundred years and did not correlate with the YD onset. (7) It was argued in several studies^13,15,18^ that individual “impact indicators” are also produced by non-impact processes, thus not requiring an impact. Finally, (8) another study^16^ argued that YD climate change had little effect on people and their animal prey across the central portion of the US. For a limited number of selected contributions relevant to this hypothesis, see Supplementary Table A3 of Wolbach *et al*.^3^

Proponents^1,10,20,21^ of the YDB impact hypothesis concede that individual proxies might have formed through alternative processes, but argue that coeval abundance peaks for the *entire suite* of proxies in the same stratum is unique to cosmic impact events. For example, the same suite of proxies has been found in the well-known Cretaceous-Tertiary (K-T) boundary layer^22,23^ (~65.5 million years ago), forming a global datum layer. Similarly, the 780,000-year-old Australasian impact event dispersed small amounts of microscopic spherules and impact glass across ~30% of the planet’s surface, forming the largest known impact strewnfield^24^, for which no physical crater has been found. As with the K-T and Australasian impact events, the YDB layer contains a suite of impact proxies within a datum layer that can also be used for global correlation and dating. Impact proponents argue that the YDB datum layer is precisely equivalent in age to the onset of the YD cooling episode recorded in the Greenland ice sheet and in many other stratigraphic sections in the Northern Hemisphere^4^. Regardless of differing hypotheses about the YDB suite’s origin, this layer forms a valuable datum for global correlation and dating.

An important new development includes the discovery by Kjaer *et al*.^25^ of a giant, 31-km-wide, possible YDB-aged impact crater under the Hiawatha Glacier in northwestern Greenland, calculated to have been produced by a 1.5-km-wide impactor. This is the 25th largest known terrestrial crater, the largest in the last ~5 million years (52-km-wide Kara-Kul crater in Tajikistan), and the second largest in the last ~35.3 million years (40-km-wide Chesapeake Bay in Virginia, USA). Impact proxies include high-temperature spherules, meltglass, amorphous carbon, and elevated concentrations of Ni, Co, Cr, PGEs (e.g., Pt, Ir, and Os), and Au^25^, all of which are also elevated at YDB sites on four continents.

Kjaer *et al*.^25^ conclude that the potential age range of the impact event spans the YD onset, although the crater is as yet undated. The potential for a YDB age is based on several lines of evidence: (1) Post-YDB annual ice layers are present from the Younger Dryas (12,800 to 11,700 cal BP) through the Holocene (11,700 cal BP to modern times), but undisturbed pre-YDB ice layers are missing. However, four widely distributed, distinctive, radar-reflecting, pre-YDB ice layers, including the youngest that spans the Bølling-Allerød (14,700 to 12,800 cal BP), are missing within the crater and within 100 km around it, consistent with having been disrupted by the impact event. (2) There is evidence of hydrothermal activity within the crater beneath the overlying 930-meter-thick ice sheet, as would be expected from residual heat generated by a young impact. If such activity had been caused by an impact event, it would be short-lived because the continuing melting of ice cover over the crater would efficiently dissipate the heat. Therefore, ongoing hydrothermal activity is consistent with a very young age for the crater. (3) The subglacial crater rim and basin appear relatively fresh and minimally eroded, consistent with a young crater^25^. If the crater were very old, the rim is expected to be much more subdued, due to the erosive effects of glacial ice movements, as is apparent with many once-subglacial Canadian crater rims that were flattened by movement of the Laurentide Ice Sheet. (4) Kjaer *et al*.^25^ report PGE anomalies in the crater derived from an ET impact event. This is similar to the results of Petaev *et al*.^26^, who analyzed Greenland ice and report an abundance peak in Pt spanning 21 years from 12,836–12,815 cal BP. They attributed this to multiple injections of Pt from a large extraterrestrial impact event, possibly now identified with the Hiawatha Crater. Kjaer *et al*.^25^ conclude that such a massive impact event “very likely had significant environmental consequences in the Northern Hemisphere and possibly globally.” If so, this impact event may have triggered climate change in southern Chile, which we explore in this contribution.

The Chilean Council of National Monuments has protected the unique Pilauco site as an important paleontological and archaeological resource because of its rich and abundant assemblage of extinct South American Pleistocene mammals and cultural remains. Prior descriptions of the stratigraphy, biostratigraphy, and chronology of this well-exposed sedimentary sequence^27–34^ provide the foundation for our investigations, and the high sedimentation rate offers high chronological resolution. Previous radiocarbon dating (9 dates^27^) has shown that the sequence includes the estimated age span of the YDB impact event (~12,835 to 12,735 cal BP)^35^. In this study, we conducted time series material analyses across the sedimentary sequence to determine changes in concentrations of Pt, palladium (Pd), high-temperature magnetic spherules, charcoal, plant macrofossils, pollen, and dung fungal spores (*Sporormiella* spp.). Also, the Pilauco stratigraphic sequence has provided an unprecedented opportunity to compare the regional megafaunal extinctions at high resolution with similar coeval extinctions in the Northern Hemisphere. We have also undertaken quantitative pollen and seed analyses across the boundary layer for paleoclimatic and paleo-environmental assessments and for comparison with YD climatic change as determined from sequences in the Northern Hemisphere.

The Pilauco site is the most extensively investigated YDB sequence south of the equator, but is also at high southern latitudes (~40°S) more than 6000 km farther south than the closest well-studied YDB site in Venezuela^5^. Identification of this YDB datum enables precise correlation between identical chronostratigraphic intervals in the Northern Hemisphere, including those at high latitudes.

**Figure 1:**
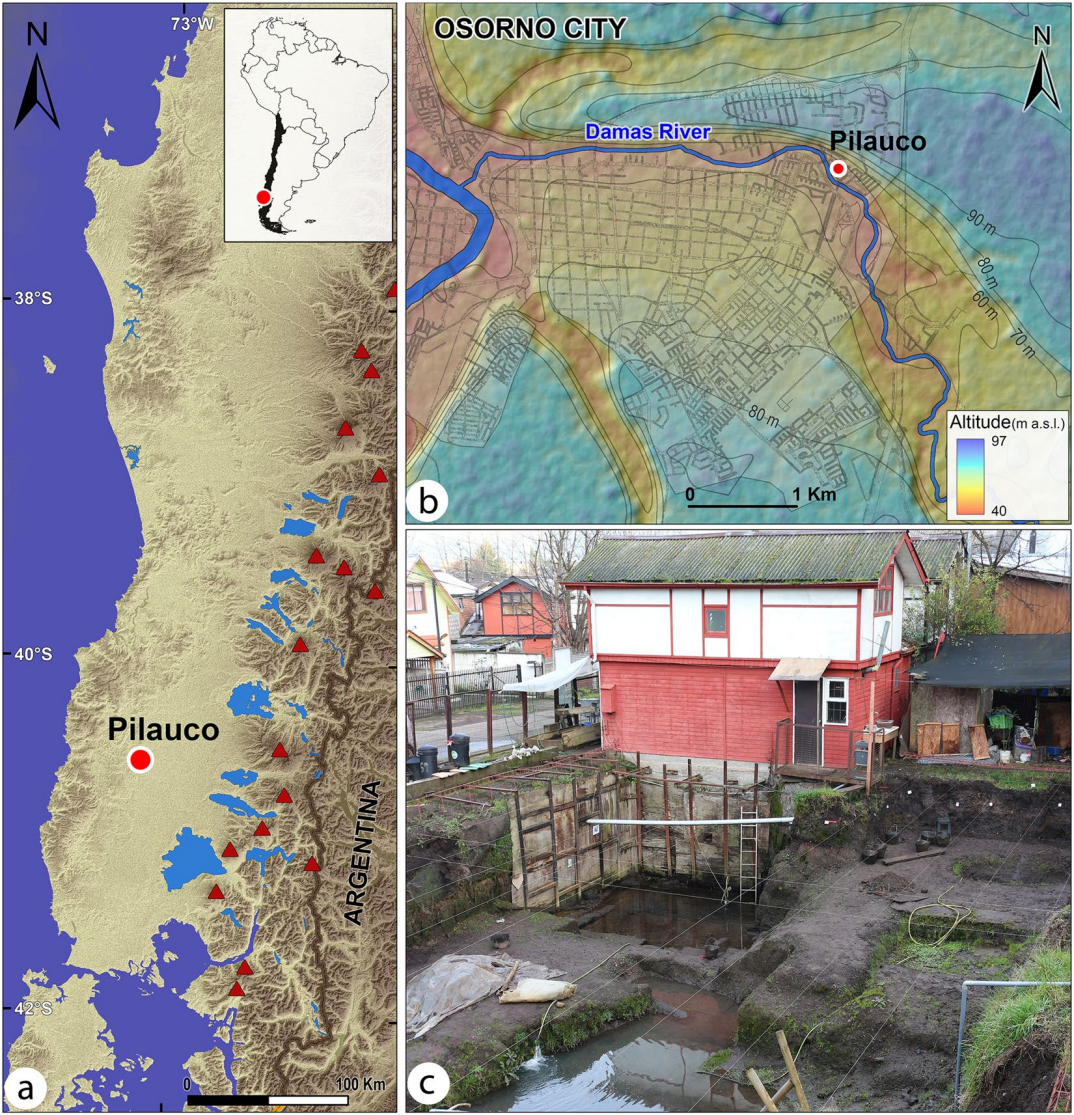
Site location. (**a**) Shaded elevation map of parts of Chile and Argentina showing regional setting of the Pilauco site; red triangles represent Andean volcanoes. Base map: ASTER GDEM is a product of NASA and METI; U.S. Geological Survey data release, 10.5067/ASTER/ASTGTM.002. Image modified with Adobe Photoshop CC2014 (https://www.adobe.com/products/photoshop.html). (**b**) Site location within the city of Osorno. Dataset for base map: ©JAXA/METI (Japan Aerospace Exploration Agency/Ministry of Economy, Trade, and Industry), 2017, 10.5067/Z97HFCNKR6VA. Both images were modified with Adobe Photoshop CC2014 (https://www.adobe.com/products/photoshop.html) and Adobe Illustrator (https://www.adobe.com/products/illustrator.html). (**c**) Photograph of the protected Pilauco site excavations.

**Figure 2:**
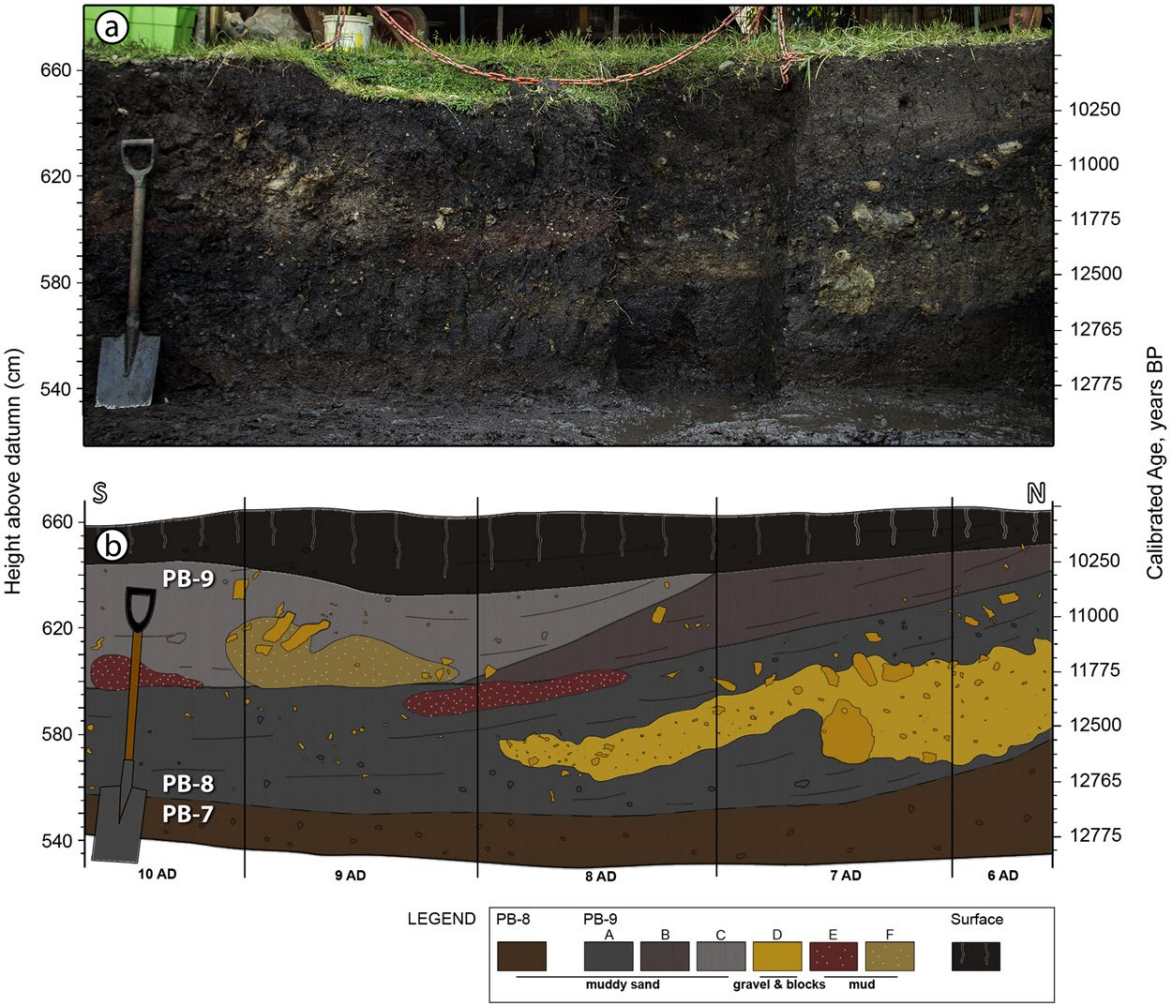
Stratigraphic section of the west wall. (**a**) West wall photograph. (**b**) Corresponding profile of grids 6AD to 10AD. These profiles show the abrupt contact between units PB-8 and PB-9, which dips ~15° to the south near the southern hill (grids 6AD to 7AD). Legend defines subunits. A young organic soil caps the sequence. See Supplementary Tables S2 and S3 for further details.

It is important to note that the main objective of this contribution is to test the YDB hypothesis and to document and discuss the wide range of evidence found within the Pilauco sedimentary section in southern Chile (Figs 1 and 2). In this study, we sought to determine whether the evidence at Pilauco is consistent or inconsistent with the YDB impact hypothesis and to explore the potential consequences of the proposed impact event.

## Section 1: Study Site

### Setting

The Pilauco site is located ~40 km west of Lakes Puyehue and Rupanco^36^ in southern Chile within the city of Osorno, northwestern Patagonia, at ~30 m above sea level (a.s.l.) (40°34′S, 73°06′W) (Fig. 1). The site presently lies in the northern part of the temperate zone of the Southern Hemisphere and annual precipitation derived from the prevailing westerly winds totals ~1330 mm, which occurs mainly during the austral fall and winter months. The mean annual temperature is 11.4 °C^37^. For details about Pilauco’s paleoclimate and biota, see Supplementary Information, S2.

### Stratigraphy and Sedimentology

The first excavations at the Pilauco site in 2007 followed the discovery there of a rich assemblage of mammalian fossils. To facilitate archaeological and paleontological investigations, the site was divided into a horizontal grid composed of 1-m^2^ elements, and the vertical sedimentary sequence was subdivided into lithostratigraphic units designated PB-6 through PB-9 (Supplementary Fig. S1).

During the last interglacial (~130,000 years ago), the Osorno area experienced intense volcanic activity from the Andean arc located ~100 km to the east (Fig. 1a). This volcanic activity deposited a series of volcanoclastic flows (50-m minimum thickness) at Secuencia San Pablo^38^, which form the hills north of Osorno and the lowermost unit at the Pilauco site. Consequently, all strata sampled at Pilauco contain abundant multi-cm-sized clasts of volcanic basalt and tuff.

The basal unit of the Pilauco stratigraphic sequence (PB-6) is an unconsolidated sedimentary bed containing abundant well-rounded pebbles and boulders. (For further details on Pilauco sedimentary stratigraphy, see Supplementary Information, S3, Supplementary Tables S2 and S3). The next higher unit (PB-7) is an organic-rich sand composed of isolated, poorly sorted colluvial volcanoclastics that contain most of the extinct megafauna remains and lithic artifacts. Unit PB-8 is composed of organic-rich mudstone separated by thin, muddy laminae, similar to unit PB-7, although it contains fewer mammal fossils and lithic artifacts than the unit PB-7 below it. Unit PB-8 also contains the youngest horse coprolite found at the site^28,39^. At Pilauco, the key stratigraphic feature is the PB-8/PB-9 boundary that marks major environmental and sedimentological changes, and is inferred from radiocarbon dating to be the YDB layer. Immediately above the YDB in unit PB-9 there is a large accumulation of wood fragments, frequently found along an undulating contact that defines a sharp lithological change (Supplementary Figs S2, S3, S4 and S5). Overall, unit PB-9 is a relatively fine-grained, black, organic-rich peat.

The charcoal-rich, peaty matrix of unit PB-9 is argued to be analogous to the “black mat” found at ~30% of the 12,800-year-old YDB sites on four continents^1,3^. The term “black mat” applies to dark, organic-rich deposits, as well as to some marls and diatomites that are white or gray, rather than black^40^. Haynes^40^ concluded that these mats are complex pedological features derived from two major processes: first, from deposition of organic-rich material, e.g., as in wetlands (29 of the 70 sites)^40–42^, and second, from soil formation possibly due to weathering of stable, organic-rich landscapes (41 of the 70 sites; see Table 2 of Haynes^40^). The classic black mat in its “type” area (the Upper San Pedro Valley of Arizona, including the Clovis site at Murray Springs) is a wetland deposit resulting from wetter conditions that occurred at and after the YD onset^40–42^. Several studies report that the local black mat is frequently older or younger than the YD onset^40–42^, but they most commonly date to the YD chronozone between ~12,800 and ~11,700 cal BP^40,41^. Most classic black mats in the United States do not contain much charcoal^40,43^, but it is sometimes abundant immediately below the black mat^40^, where the YDB layer typically is found^1,3^ Across Northern Europe, layers analogous to black mats in the United States are typically rich in charcoal^1^.

Wolbach *et al*.^3^ summarized previous studies (their Table A2) showing that 24 of 27 YDB sites with black mats also display peaks in YDB proxies (e.g., magnetic spherules, carbon spherules, high-temperature meltglass, and/or nanodiamonds). The authors of these studies argued that the presence of these YDB proxies suggests, but is not conclusive of a relationship among the YDB impact event, an intense episode of biomass burning, and the 12,800-year-old black mat.

After the black mat’s discovery at Pilauco, several stratigraphic sections on the west wall of the excavations were sampled for different environmental and potential impact-related proxies. Grid 8AD samples were examined for seeds, magnetic spherules, and Pt concentrations (Supplementary Fig. S2). Samples from grid 10AD were examined for charcoal and pollen concentrations (Fig. 2), and grid 14AD was sampled for radiocarbon dating and *Sporormiella* spp. spores (Supplementary Fig. S3). All grids were examined for megafaunal remains, wood fragments, and lithic artifacts^28^. The potential presence at Pilauco of meltglass, carbon spherules, glasslike carbon, and other reported YDB proxies has yet to be investigated. Experiments are underway to investigate the presence of nanodiamonds and aciniform carbon/soot that often are associated with cosmic impact events^1,3,11^. In the Methods section below those workers responsible for sample collection and testing are indicated.

## Section 2: Chronology

**Figure 3:**
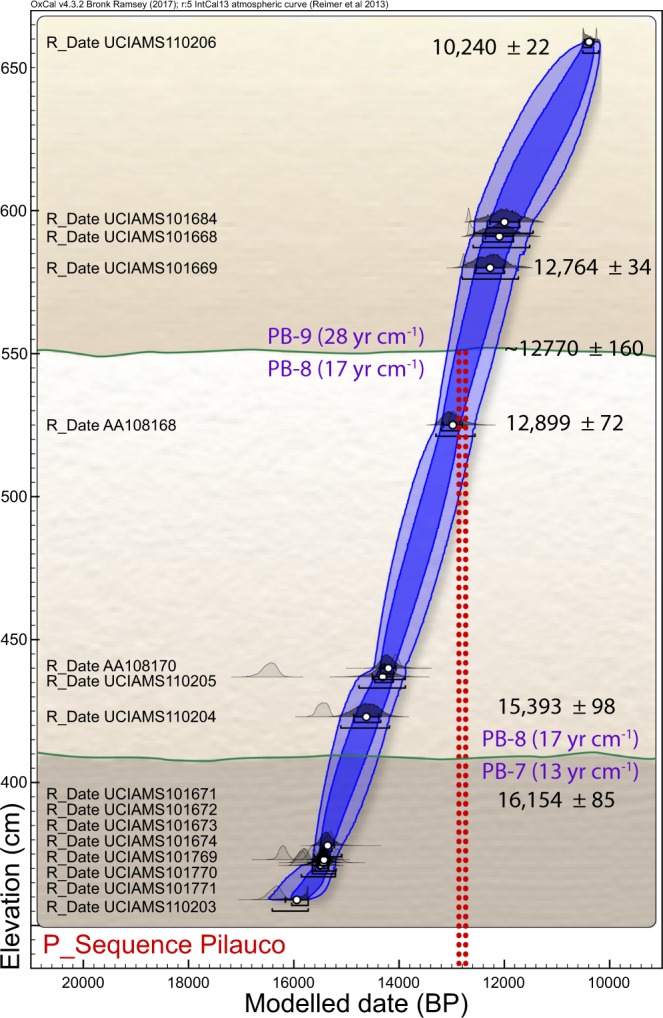
Bayesian age-height model based on 16 radiocarbon dates from grid 14AD (Supplementary Fig. S1 and Table S5). Green lines represent the stratigraphic boundaries PB-7/PB-8 and PB-8/PB-9; the latter has a Bayesian-calculated age of 12,770 ± 160 cal BP. Inferred depositional rates in yr cm^−1^ are in parentheses. Vertical red dotted lines represent age range of 12,835–12,735 cal BP for the proposed YDB impact event^35^. Red dotted lines overlap the PB-8/PB-9 boundary, consistent with the age of the YDB cosmic impact event. Produced with OxCal v. 4.3.2, SHCal13 calibration curve for the Southern Hemisphere.

In developing an age-height model (Fig. 3), we used Bayesian analysis based on the protocol given in Kennett *et al*.^35^. This age-height model allows us to infer the following Bayesian age spans and deposition rates for the sedimentary profile in grid 8AD. PB-6: this unit contains wood fragments with a youngest calibrated radiocarbon age of 17,233 ± 103 cal yr BP (Supplementary Tables S4 and S5). Bayesian analysis indicates the boundary of PB-6 (~325 cm) dates to ~16,260 ± 260 cal yr BP with a terminal deposition rate of 19 yr cm^−1^, the slowest rate observed in the profile. PB-7: the age span of this unit is ~16,260 ± 260 (at ~325 cm) to 15,200 ± 240 cal yr BP (405 cm) with an average deposition rate of 13 yr cm^−1^. PB-8: the age span of this unit is 15,200 ± 240 cal yr BP (405 cm) to 12,770 ± 160 cal yr BP (550 cm, representing the YDB layer) for an average deposition rate of ~17 yr cm^−1^. The sedimentation rate across the PB-8/PB-9 boundary is ~5 cm in ~2.5 years, a very rapid rate of ~0.5 yr cm^−1^, the fastest observed in the sequence. This unit represents a continuous record that allows high-resolution investigations such as ours. PB-9: this unit ranges in age from ~12,770 ± 160 (550 cm) to 10,250 ± 150 (640 cm) with an average deposition rate of ~28 yr cm^−1^.

## Results and Discussion

### Section 3: Spherulitic sedimentary particles

At Pilauco, we identified several discrete groups of rounded micro-particles (Supplementary Table S6) that include, (1) melted Fe- and Si-rich spherule, (2) melted chromium (Cr)-rich spherules, (3) melted Si-rich volcanic spherules, (4) non-melted authigenic framboids, (5) melted anthropogenic spherules, and (6) non-melted detrital grains that resemble spherules.

#### Group 1: YDB spherules

**Figure 4:**
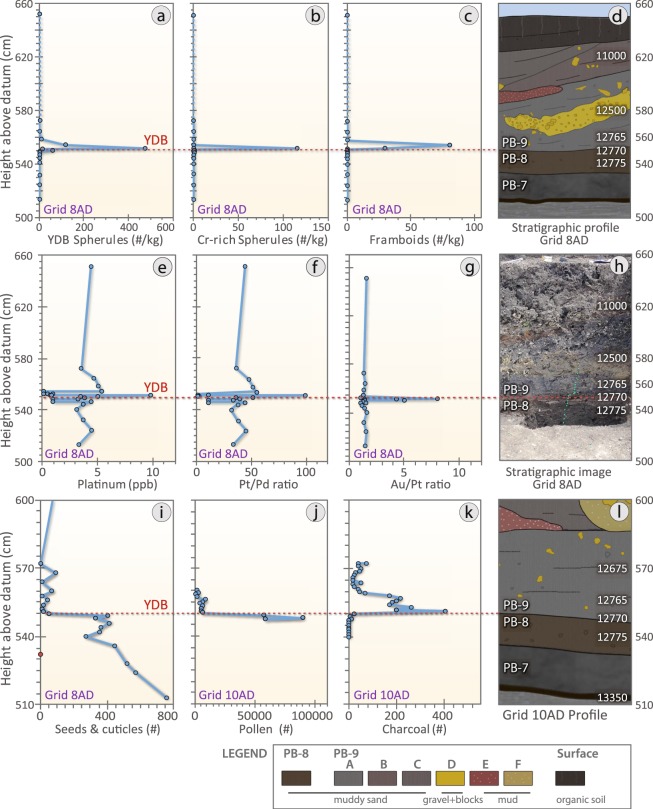
Changes in impact-related and environmental proxies at the PB-8/PB-9 boundary (YDB), showing peak concentrations of high-temperature impact spherules, framboids, and elemental proxies, including Pt, along with changes in charcoal and plant macrofossil abundances. (**a**) Group 1: high-temperature, Fe- and Si-rich impact spherules exhibit a peak abundance of ~520/kg at ~552 cm; (**b**) Group 2: Cr-rich spherules have a peak abundance of ~115/kg at ~552 cm; (**c**) Group 4: authigenic framboids have a peak abundance of ~80/kg at ~554 cm. (**d**) Stratigraphic profile of grid 8AD. Age of PB-8/PB-9 boundary is 12,770 ± 160 cal yr BP. (**e**) Pt abundance peak of 9.9 ppb in the YDB layer at 551 cm coincident with the boundary of units PB-8 and PB-9. (**f**) Anomalous Pt/Pd ratios are restricted to the PB-8/PB-9 boundary suggesting the influx of non-local Pt at the YDB. (**g**) Anomalous Au/Pt ratios are also restricted to the PB-8/PB-9 boundary suggesting the influx of non-local Au at higher concentrations than those of the non-local Pt. (**h**) Photograph of sampling section in grid 8AD. Green pins mark sampling levels. (**i**) Abundance record of seeds showing the major decline at the YDB layer. (**j**) Pollen concentrations showing the abrupt decline in the YDB layer. (**k**) Abrupt increase in charcoal peaking at the YDB layer with continuing high concentrations in the overlying ~10 cm. (**l**) Stratigraphic profile for grid 10AD. Group 3 volcanic spherules were found in low concentrations throughout the profile in grid 8AD (not plotted here; see Supplementary Fig. S11a). Group 5 anthropogenic spherules were restricted to surface sediments in grid 8AD (not plotted here; see Supplementary Fig. S11b). Group 6 rounded detrital grains (not plotted) are common throughout the entire profile. Legend is the same as in Fig. 2.

**Figure 5:**
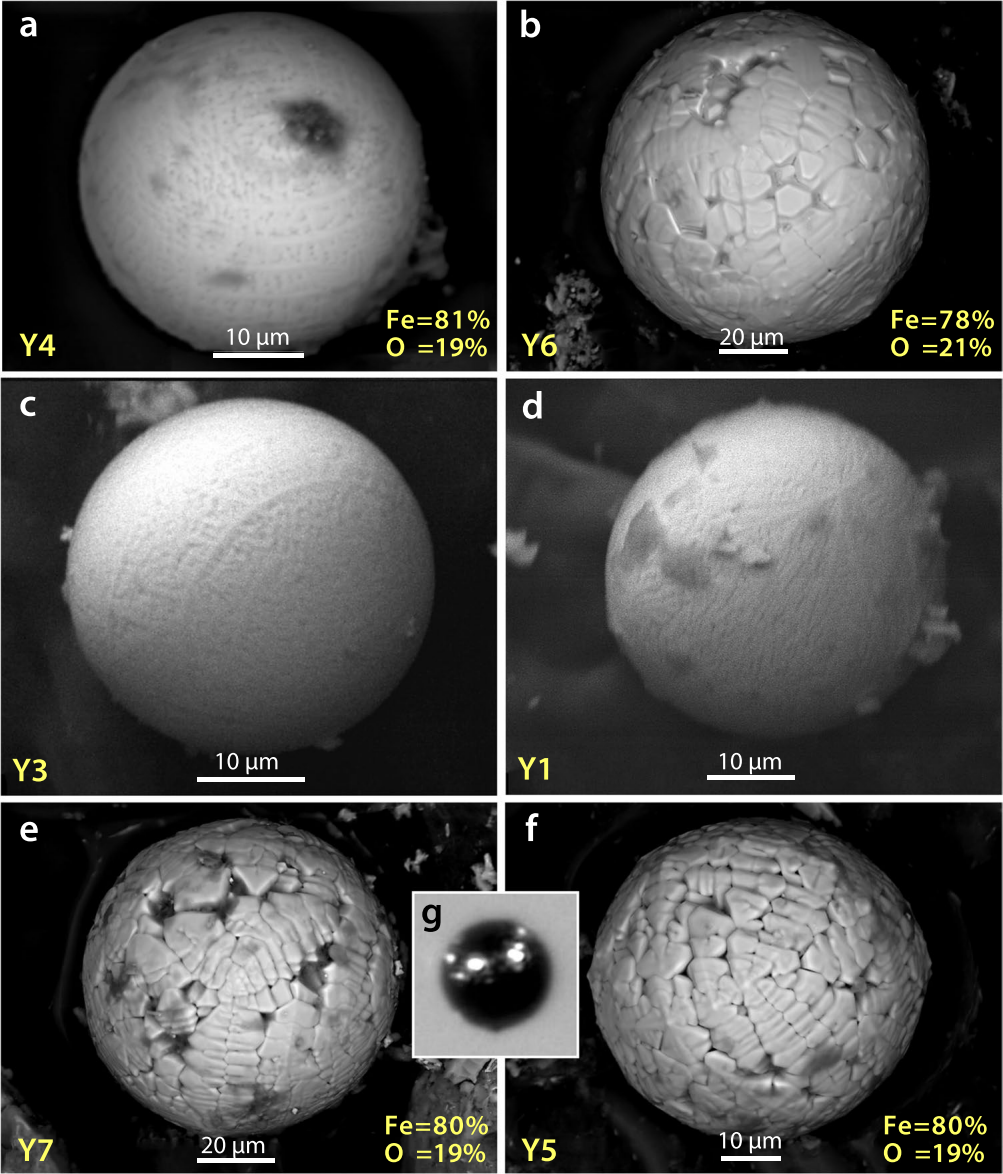
Group 1 SEM Images: High-temperature Fe-rich impact-related spherules. (**a**–**f**) High-temperature impact spherules have distinctive dendritic surface texturing indicative of rapid quenching from melt temperatures above ~1450 °C. SEM-EDS analyses for each spherule are in Supplementary Table S7. Elemental composition of spherule in (**b**) is in Supplementary Fig. S6. (**g**) Photomicrograph of high-temperature impact-related spherule illustrating the difficulty in distinguishing these from framboids (Fig. 11a**)** and detrital grains (Fig. 11c). Note percentages of Fe and O at lower right of some images. The composition of these spheroids is FeO (wüstite), a highly reduced mineral that almost never occurs under natural terrestrial conditions, but is common in meteorites and materials produced during impact events under oxygen-deficient conditions.

Previous studies^1,10,20,44–46^ have identified distinctive crystalline textures on Fe-rich YDB spherule surfaces, indicative of high-temperature melting at >1450 °C followed by rapid quenching. In grid 8A at Pilauco, an abundance peak in melted, textured YDB spherules was observed only in samples between 580 and 550 cm (Fig. 4a,d), and was not found in other samples. The highest measured concentration of impact-related spherules at Pilauco is ~520/kg (Fig. 4a) at 552 cm in Grid 8AD (Fig. 4d). The average size of these spherules is ~45 µm with a range of ~95 to ~10 µm (Fig. 5 and Supplementary Fig. S6).

Eleven SEM-EDS analyses show that Group 1 spherules are distinctive in that they contain only two oxides: FeO, averaging 93.7 wt.% (range: 100 to 86.6 wt.%) with a small percentage of SiO_2_ (Supplementary Table S7). High formation temperatures for these spherules are indicated by the presence of FeO (melting point: ~1450 °C), SiO_2_ (melting point: ~1750 °C) and a lack of low-temperature volatiles such as Na_2_O and K_2_O, which vaporize at lower temperatures. The presence of titanium in some of Pilauco’s YDB spherules is consistent with melting of titanomagnetite grains as has been previously reported^44,47^. We infer that some Group 1 spherules formed from the melting of magnetite and titanomagnetite grains that are common in the Pilauco bulk sediments.

**Figure 6:**
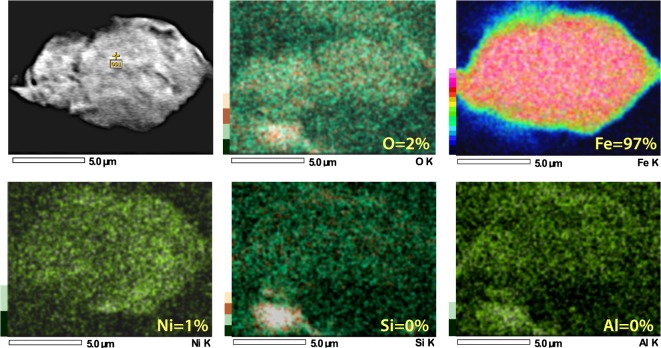
SEM-EDS elemental map of YDB magnetic grain. Upper left, SEM image of magnetic grain. SEM-EDS elemental map and separate spot analysis (yellow cross on upper left image) show that this highly reduced grain is composed of ~97% Fe, 2% O, and ~1% Ni, suggesting a possible extraterrestrial origin. Si and Al were undetectable in the grain itself but were detected in surface debris shown by the whiter-colored areas in the two lower right images. Elemental composition is provided in Supplementary Fig. S7.

Group 1 spherules found at Pilauco are unusual in that they exhibit considerable variation in composition and oxygen fugacity, ranging from oxygen-reduced, dendritic, Fe-rich spherules to fully oxidized Fe-oxide spherules that are found in very close proximity to one another. Four Group 1 spherules (Fig. 5a,b,e,and f), for example, were formed from highly reduced melt with a low oxygen fugacity (*f*O2) along the Fe-wüstite buffer (IW) (Supplementary Table S7). These spherules are not composed of typical terrestrial Fe_2_O_3_ (hematite; Fe:O ratio of ~70:30) or Fe_3_O_4_ (magnetite; Fe:O ratio of ~72:28), but rather of FeO (wüstite; Fe:O ratio of ~78:22 to ~81:19). This composition rarely occurs under normal terrestrial conditions (e.g., from volcanism or anthropogenesis), but is common in meteorites and impact melts having much lower oxygen percentages. In addition, some non-spherulitic magnetic grains extracted from the YDB layer (552 cm) are composed of highly reduced native Fe at 97% Fe (Fig. 6, Supplementary Fig. S7) and/or reduced magnetite as FeO (Supplementary Fig. S8). Similar low-oxygen melted minerals were observed in spherules and meltglass from the YDB layer at Abu Hureyra, Syria and in meltglass from the Trinity atomic bomb test site at Alamogordo, New Mexico^20^.

**Figure 7:**
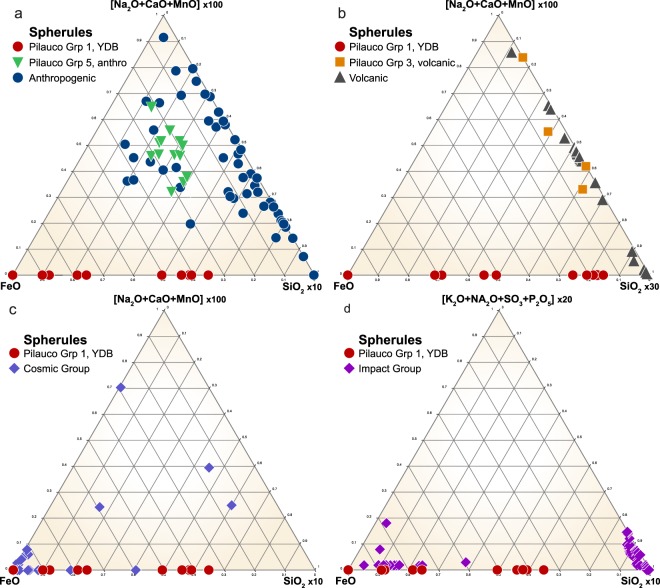
Ternary diagrams comparing compositions of spherules from the Pilauco site with other known types of spherules described in the literature^20,44,46,65^. (**a**) Group 1 high-temperature Fe-rich spherules (red dots) are compositionally unlike known anthropogenic spherules (blue dots) and unlike Group 5 anthropogenic spherules (green triangles) found at the surface of the profile in grid 8AD. (**b**) Group 1 spherules are compositionally different from known volcanic spherules (black triangles), which are geochemically similar to Group 3 Pilauco spherules (orange squares) thought to be volcanic in origin. (**c**) Group 1 spherules are geochemically similar to some cosmic spherules (blue diamonds) indicating that some YDB layer spherules at Pilauco might be cosmic in origin. (**d**) Group 1 spherules also are compositionally similar to spherules produced as ejecta during known cosmic impact events. Comparative data of non-Pilauco material are from Table 5 in the supplementary materials of Bunch *et al*.^20^.

By plotting the SEM-EDS results of Pilauco Group 1 high-temperature Fe-rich spherules in ternary diagrams (Fig. 7), comparisons can be made to other known spherule types. Group 1 spherules are dissimilar in composition to known anthropogenic spherules that are produced primarily by coal-fired power plants, and are typically enriched in CaO, MnO, and low-temperature Na_2_O^20,46^ (Fig. 7a). Group 1 spherules are also dissimilar to known volcanic spherules (Fig. 7b), which are typically more highly enriched in CaO, MnO, as well as low-temperature Na_2_O^20,46^. Additionally, the presence of rapidly quenched Fe crystals (Fig. 7a), requiring high temperatures >1450 °C, rules out an authigenic origin because, by definition, such spherules are unmelted.

The ternary diagrams also indicate that Group 1 spherules do not have typical meteoritic compositions because most stony micrometeorites (96% of all micrometeorites) have MgO concentrations >10 wt.%^20,46^ whereas Group 1 spherules contain no detectable MgO. In addition, Group 1 spherules are not Fe-rich micrometeorites ablated from typical Fe meteorites (~4% of meteorites) because such meteorites typically have high-Ni concentrations (averaging 9.0 wt.%, n = 76; maximum = 42.6 wt.%^48^) whereas Group 1 spherules contain no detectable Ni. Pilauco’s Group 1 spherules, however, could have been ablated from a low-Ni, low-MgO impactor. In addition, Group 1 spherules are geochemically similar to spherules produced during extraterrestrial impact events that melted and ejected target rocks (Fig. 7d). Impact-related spherules from a number of confirmed sources fall into two distinct geochemical groups, one that is Fe-rich and the other Si-rich; the compositions of the YDB spherules from Pilauco only overlap with those of the former.

#### Group 2: Chromium-rich spherules

**Figure 8:**
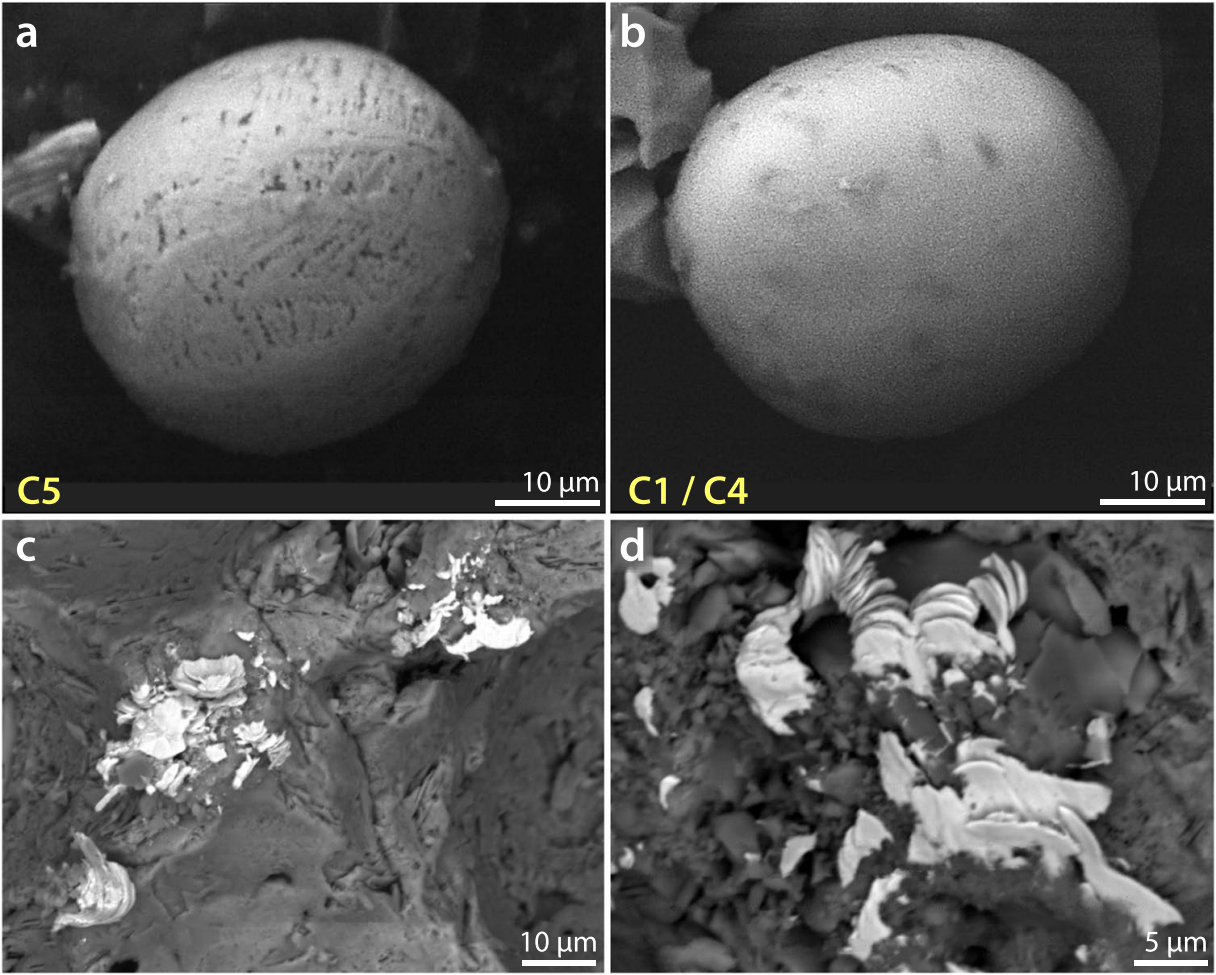
Group 2: Cr-rich spherules from Pilauco. Micrographs (**a**,**b**) show Cr-rich ovoidal spherules with SEM-EDS compositions matching Cr-magnetite inclusions that are commonly found in basaltic glass distributed randomly throughout the Pilauco profile. The spherule in (**a**) displays distinctive dendritic texturing indicative of melting and rapid quenching at temperatures above ~1670 °C, the melting point of Cr-magnetite. SEM-EDS compositional data for the spherules shown in Supplementary Table S7. (**c**,**d**) Typical Cr-magnetite inclusions in basaltic glass from Pilauco at a height of 513 cm that are commonly found in all samples throughout the profile. Scalloped stack of individual Cr-rich inclusions most likely resulted from supergene or hypogene processes (deposition or enrichment of mineral deposits by solutions moving downward through magmatic rocks).

A second group of spherules was also found only in the 12,800-yr-old layer at Pilauco (552 cm). This group shows a concentration of ~115 spherules/kg ranging from ~45 to ~42 µm in diameter with an average of ~43 µm (Figs 4b and 8a,b; Supplementary Table S6). Group 2 is differentiated from Group 1 spherules by the presence of Cr oxide (Cr_2_O_3_) that averages 6.2 wt.% (range: 3.9 to 13.3 wt.%) (Supplementary Table S7). Approximately half of these Cr-rich spherules contain TiO_2_ (average: 6.1 wt.%; range of 8.6 to 0.0 wt.%), and no other Pilauco spherules observed outside of the 12,800-yr-old layer contain any detectable Cr_2_O_3._

In exploring the source of the Cr-rich spherules, we determined that all 23 sediment samples from 651 to 513 cm contain variable amounts of Cr-rich vesicular basalt fragments that are up to several cm in diameter. In one sample, the basaltic matrix is composed of aluminosilicate glass (Al: 2.3 wt.%; Si: 19.1 wt.%; Mg: 8.1 wt.%; and Ca: 8.6 wt.%) (Supplementary Fig. S9a). Although the bulk sediment contains no detectable Cr, those basaltic fragment examined contain numerous Cr-magnetite inclusions (Cr: 10.1 wt.%; Fe: 74.8 wt.%; Si: 5.5 wt.%; and Al: 2.6 wt.%) (Fig. 8c,d; Supplementary Figs S9b and S10) that most likely crystalized as the lava cooled. These non-spherulitic inclusions contain nearly identical relative concentrations of Cr and Fe as those found in the Cr-rich spherules of Group 2 (Cr: 10.1 wt.%; Fe: 74.8 wt.%; Si: 5.5 wt.%; and Al: 2.6 wt.%) (Supplementary Table S7) suggesting that the Cr-rich spherules formed from the melting of local Cr-rich basaltic material.

The Cr-rich spherules from Pilauco contain an average of 6.05 wt.% Cr_2_O_3_ (max: 8.58 wt.%), and are unlike any known volcanic spherules, including those from two Peruvian volcanoes (Huainaputina and Ubinas) that contain an average of only 0.05 wt.% Cr_2_O_3_ (range of 0.00 wt.% to 0.73 wt.%)^49^. Similarly, there are no known volcanic spherules reported in the literature that contain more than ~1.1 wt.% Cr_2_O_3_^49^, which is much less than the minimum of 3.9 wt.% found in the Cr-rich spherules from Pilauco. Group 2 spherules also contain high concentrations of Fe unlike other known volcanic spherules, which are typically composed of FeO averaging 12.2 wt.%, SiO_2_ averaging 50.9 wt.%, Al_2_O_3_ averaging 18.5 wt.%, and CaO averaging 10.0 wt.%. The maximum amount of Fe reported in volcanic spherules is 35.5 wt.%, compared to the minimum amount of 74.3 wt.% in Pilauco’s Cr-rich spherules.

After a volcanic eruption, Cr-magnetite inclusions can form from supergene or hypogene processes (deposition or enrichment of mineral deposits by solutions moving downward through magmatic rocks)^50^ at ambient temperatures of far less than that of molten basalt^51^ (~1300 °C). Much higher temperatures of ~1670 °C to 2160 °C, are required to re-melt these Cr-rich inclusions^52,53^ to form spherules. Because this range exceeds known temperatures of erupting magma, the melted Cr-rich spherules at Pilauco almost certainly were produced by some post-eruptive, non-volcanic process. The high temperatures required to produce the Cr-rich spherules are limited to only a few processes, such as anthropogenesis, lightning, and cosmic impact^20^. Anthropogenesis seems unlikely because we found no similar Cr-rich spherules at the surface in industrial-age sediments, but only at a depth of ~100 cm (height of 552 cm) in the ~12,800-year-old YDB layer. Lightning strikes are also an unlikely source because such spherules should therefore be a common occurrence in all strata, but instead, are restricted only to the 12,800-year-old YDB layer. This leaves cosmic impact as the most likely origin.

#### Group 3: Ca- and Si-rich basaltic volcanic spherules

**Figure 9:**
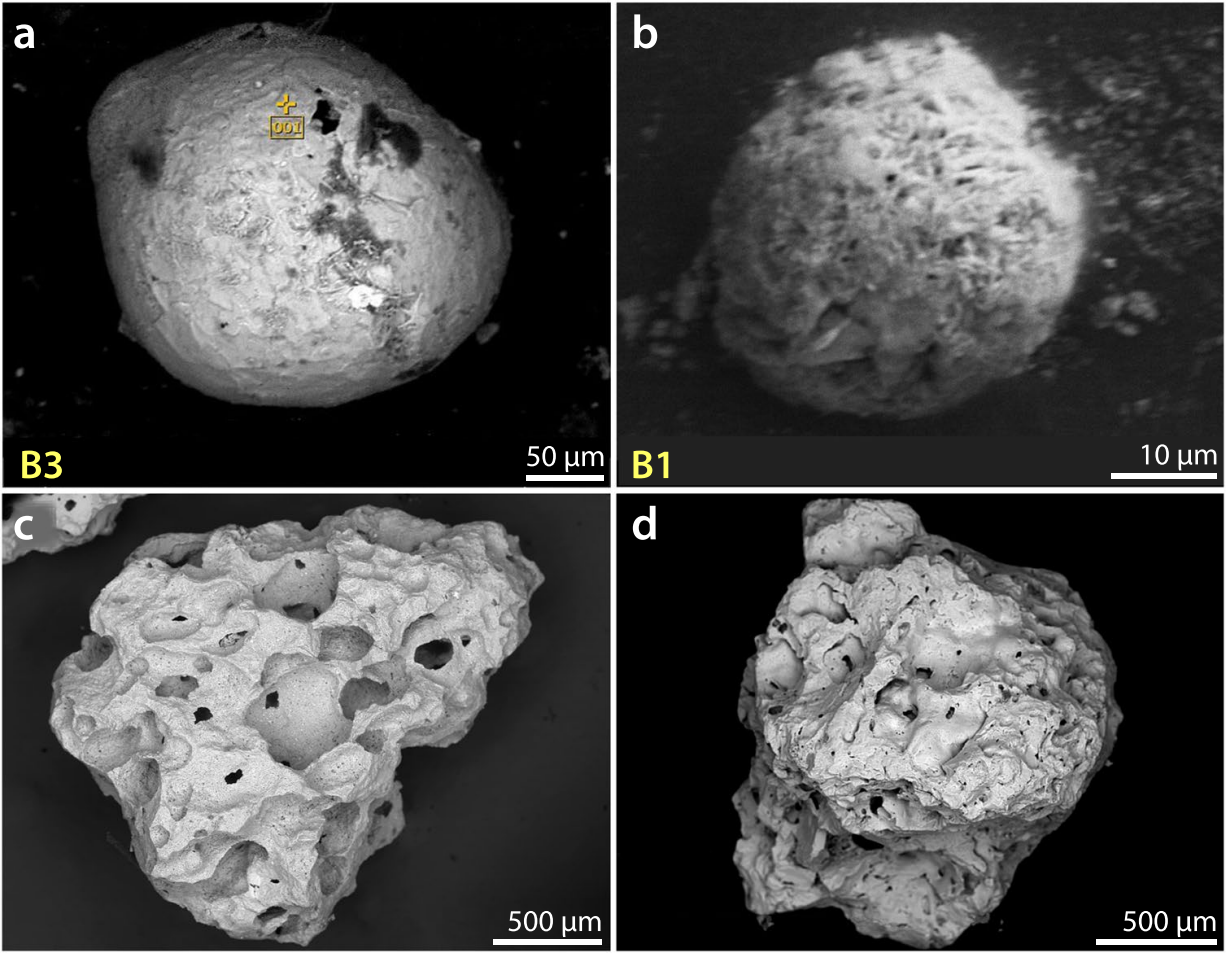
Ca- and Si-rich basaltic volcanic spherules and basaltic glass fragments from Pilauco. (**a**,**b**) Group 3 volcanic spherules contain SiO_2_ at >17 wt.% and SO_3_ at >1 wt.%, but lack Cr_2_O_3_. Compositions are listed in Supplementary Table S7. (**c**,**d**) Vesicular basaltic glass fragments from a height of 548 cm that are commonly found in all samples from the Pilauco profile. These fragments of basaltic glass contain Cr-rich inclusions and are geochemically similar to the Group 3 volcanic spherules in (**a**,**b**).

A third group of spherules are Ca- and Si-rich relative to Group 1 and 2 spherules and are randomly distributed at low concentrations (0 to ~10 spherules/kg) in the five sedimentary samples analyzed from Pilauco (Fig. 9a,b, Supplementary Fig. S11a). None were found in the YDB layer. These spherules contain a range of ~61 to 17 wt.% SiO_2_ along with highly variable concentrations of Na_2_O, MgO, SO_3_, and CaO (Supplementary Table S7). These compositions closely match those of the vesicular basalt fragments found in Pilauco sequences (Supplementary Fig. S5a), suggesting they are of volcanic origin.

Volcanic spherules like those of Group 3 are produced during eruptions of low-viscosity magmas when micro-droplets form that have the same composition as the melt^54^. Spherules from low-energy eruptions do not fall far beyond the base of volcanoes, which includes the foothills of the Andes near Pilauco. Volcanic eruptions capable of distributing tephra across wide areas are violent, explosive events driven by high gas pressures^55^, and these energetic eruptions produce tephra shards that are angular rather than rounded. For instance, the largest known eruption of the last 5 million years, the 75,000-year-old Toba-lake eruption distributed tephra up to 2500 km from the caldera, but did not produce any known spherules^55^. Hence, it is unlikely that volcanism can account for spherules in the YDB layer at Pilauco (Group 1) as well as those found at other YDB sites on other continents^20,46^. Furthermore, there are no reported examples of unequivocal volcanic spherules that contain 85 wt.% to 100 wt.% FeO as found in the Group 1 YDB spherules from Pilauco and from other YDB sites. Magma can contain Fe-rich crystals that can crystallize from the melt slowly over time, but there is no known mechanism by which YDB-like spherules containing >85 wt.% FeO can form from erupting magma composed of no more than ~13 wt.% Fe.

#### Group 4: Authigenic framboidal spherules

A peak in spherulitic framboids (~80/kg) occurs at a height of 554 cm, ~2 cm higher than the high-temperature YDB spherules found at 552 cm (Fig. 4c). These spherules are predominantly composed of hundreds of unmelted, cube-like, pyrite crystals (FeS_2_) that formed slowly under anoxic conditions rather than instantaneously as with melted impact-related spherules^46^. Even though framboids are not directly related to the impact event, they are commonly associated with high-temperature impact-related spherules in the YDB layer at other sites^10,20,44,46^, possibly because of post-YDB environmental disruption.

#### Group 5: Anthropogenic spherules

**Figure 10:**
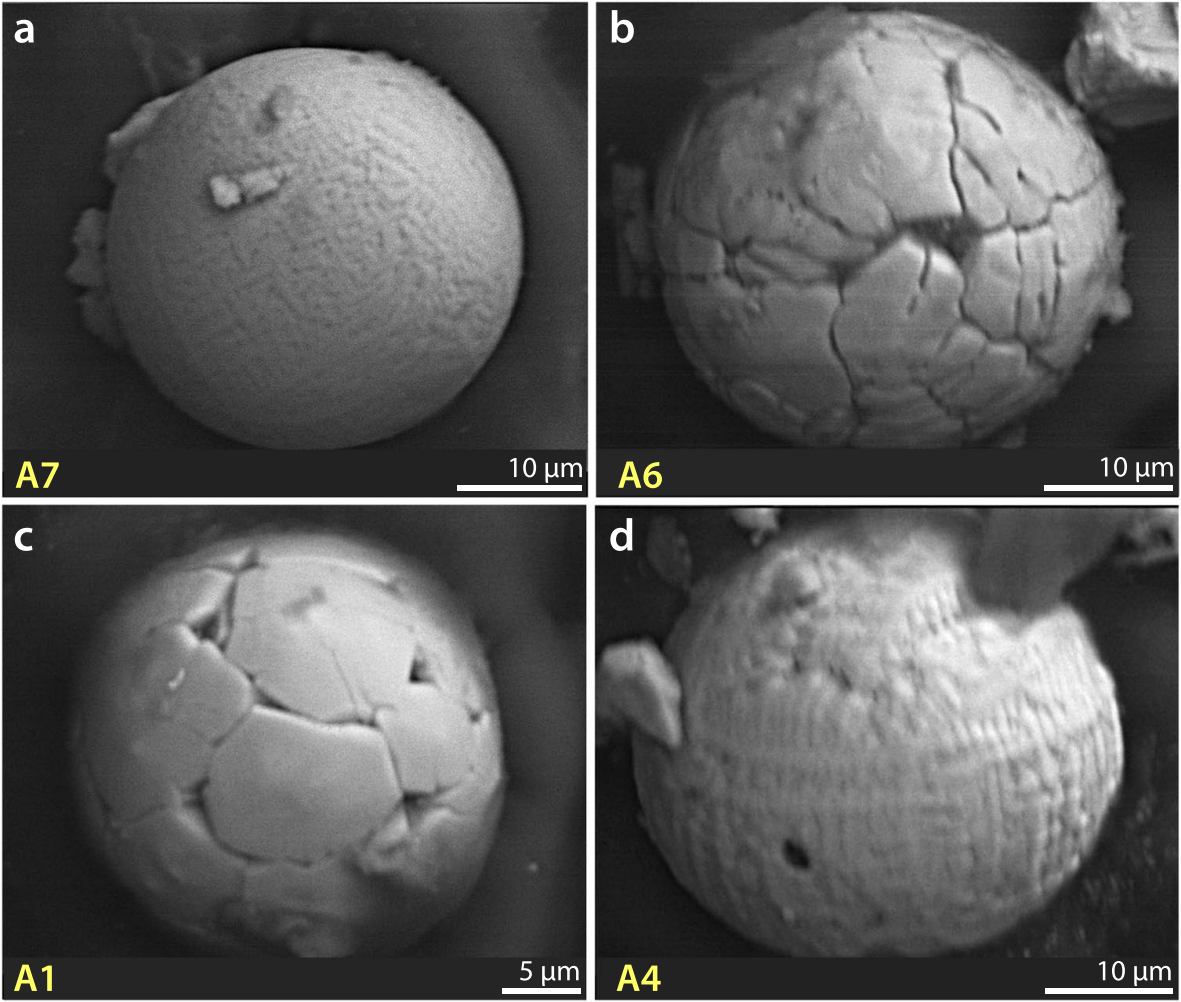
Group 5: Anthropogenic spherules. (**a**–**d**) SEM images of anthropogenic spherules found only in surface sediments at Pilauco. They are morphologically similar to Group 1 impact-related spherules, but SEM-EDS analyses show that these spherules are geochemically dissimilar to all other groups at Pilauco. Ternary diagram in Fig. 7a demonstrates geochemical similarity of them to known anthropogenic spherules.

Spherules in Group 5 were found only in surface, industrial-age sediment samples at a height of 651 cm with an abundance of ~440 spherules/kg (Fig. 10, Supplementary Fig. S11b). Because these spherules are dendritic-textured, and hence morphologically similar to impact-related spherules of Group 1, they can only be differentiated geochemically by using SEM-EDS. The composition of these spherules closely matches known anthropogenic spherules (Fig. 7a), which are typically produced in large quantities by industrial processes, often from the burning of coal and combustion-related melting of included magnetite grains^56^. Known anthropogenic spherules contain uniquely distinctive concentrations of MnO, MgO, CaO, and Al_2_O_3_, typically at ≤1 wt.%^20^, as we observe in Group 5 spherules from Pilauco. Puffer^57^ examined spherules from a wide variety of settings and concluded that anthropogenic spherules frequently contain 0.4–0.8 wt.% Mn, which is commonly used in the smelting of ferromanganese alloys. These values are similar to the range of 0.2 wt.% to 0.9 wt.% (average: 0.3 wt.%) for MnO in Group 5 spherules from surface samples at Pilauco.

#### Group 6: Rounded detrital grains

**Figure 11:**
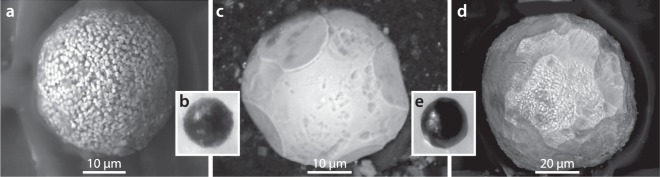
Group 4: Authigenic pyritic framboids and detrital magnetite. (**a**) SEM image of a framboid that contains Fe, sulphur, and oxygen and is composed of distinctive, unmelted cube-like crystals that formed slowly over time. (**b**) Photomicrograph of a framboid showing how difficult it is to distinguish these from YDB spherules (Fig. 5g) and detrital grains (**e**) using reflected-light microscopy. (**c**,**d**) Sub-rounded detrital grains rich in TiO_2_ (average: ~36 wt.%) and FeO (average: 64 wt.%). (**e**) Photomicrograph of detrital grain showing its similarity to YDB spherules (Fig. 5g) and framboids (**b**) emphasizing the need to use SEM-EDS analysis.

Throughout the Pilauco section, we observed detrital titanomagnetite grains (Fig. 11c–e) that when rounded and smoothed by abrasion can be easily mistaken for impact-related spherules when using reflected-light microscopy. The difficulty in differentiating spherule groups is evident in the photomicrographs of impact-related spherules (Fig. 5g), framboids (Fig. 11b), and detrital grains (Fig. 11e).

#### Summary of independent YDB spherule investigations

This paper describes in detail multiple types of spherulitic objects including authigenic, volcanic, detrital, framboidal, and YDB spherules, and Supplementary Table S8 summarizes a number of the unique and distinguishing characteristics of each type. Numerous other investigations^1,10,20,44,46^ have shown that these various particles cannot be differentiated using reflected-light microscopy, but require the use of SEM-EDS as originally specified by Firestone *et al*. (page 17–18 of their Supporting Information)^1^. Of 13 subsequent independent studies, all claimed to have followed the Firestone protocol (details of studies in Supplementary Information, S1), but only eight studies correctly performed SEM-EDS analyses, and all eight confirmed the results of Firestone *et al*.^1^. Five of the 13 studies^15,43,58–60^ reported that “YDB spherules” are distributed in non-YDB layers throughout the sediment investigated, and therefore, cannot be impact-related. However, those five studies either did not conduct SEM-EDS at all or did not correctly differentiate YDB spherules from non-YDB spherulitic objects, such as volcanic spherules, framboids, and detrital grains. These disparate results, some from studies using the same sedimentary profiles, clearly emphasize the necessity of performing SEM-EDS analyses.

### Section 4: Extraterrestrial platinum (Pt)

**Figure 12:**
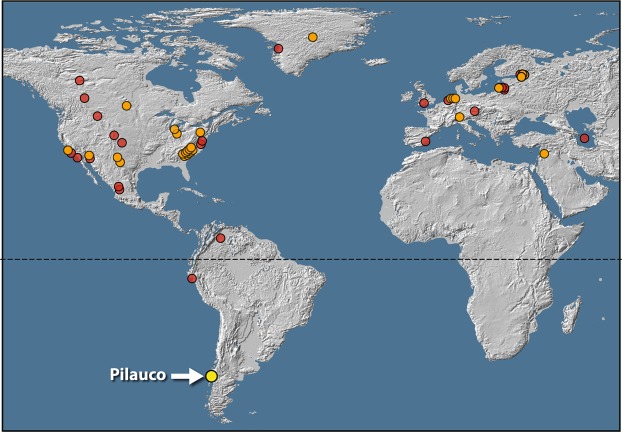
Location map showing 53 YDB sites. Orange dots represent 28 sites with peaks in both platinum (Pt) and other impact proxies such as high-temperature Fe-rich spherules. Red dots represent 24 sites with impact proxies but lacking Pt measurements. Yellow dot indicates the Pilauco site. North and Central America, n = 30 sites; South America, n = 3; Greenland, n = 2; Europe, n = 16; and Asia, n = 2. Map source: USGS, Sioux Falls, accessed October 17, 2011; and Japan ASTER Program (2003), ASTER Global Digital Elevation Map, GDEM-10 km-BW, accessed October, 2017 from https://asterweb.jpl.nasa.gov/gdem.asp, 10.5067/ASTER/ASTGTM.002. Modified with Adobe Photoshop CC2014 (adobe.com/products/photoshop.html).

The Pt-group elements (Pt, Ir, and Os) are common constituents of meteoritic and cometary material and are often used as indicators of extraterrestrial impact events. In their study of the YDB event, Petaev *et al*.^26^ identified a conspicuous peak in Pt abundance at the onset of YD climate change in a Greenland ice core (Greenland Ice Sheet Project 2). These authors attributed a decades-long episode of Pt deposition to a cosmic impact event that resulted in “multiple injections of Pt-rich dust into the stratosphere^26^”. The existence of such an impact proxy at the YD onset was predicted for Greenland ice core records at the time the YDB hypothesis was first proposed^1^. Since that initial discovery of a YDB Pt peak in Greenland, several more investigations have found elevated concentrations of Pt, and other Pt-group elements (PGEs), and high-temperature PGE-rich spherules, marking the YD onset within 28 widely separated sediment sequences across North America, Europe, and Asia^45,47,61–64^ (Fig. 12). Prior to the present investigation, no YDB-age enrichments in Pt have been reported for YDB sites in South America.

At Pilauco we measured Pt, Pd, and gold (Au) concentrations in 17 samples, plus eight duplicates, using fire-assay and inductively-coupled-plasma mass spectrometry (ICP-MS). The results show that one significant Pt peak (Fig. 4e) in grid 8AD (Fig. 4h) reached 9.9 ppb at 551 cm (Supplementary Table S9). This value, the highest concentration in the record, is more than 3 × higher than the average background values of 2.7 ppb (range: 0.1 to 5.4 ppb) and closely corresponds with the peak in high-temperature YDB impact spherules. Two other aliquots of the same sample contained 0.7 and 0.8 ppb of Pt, values that are below background levels for unknown reasons. Perhaps the rapid deposition diluted Pt in the sediment and/or the absence of high-concentration Pt nuggets reduced the average concentrations^1,3,45,65^. Similarly, both Pd and Au reach large abundance peaks at 551 cm (Supplementary Fig. S12a,b).

After normalizing Pt to Pd, we found that the Pt/Pd ratios are 2× higher than background ratios (Fig. 4f) in the YDB layer, but not in samples from above or below it. Similarly, Au/Pt ratios are 5× higher than background ratios (Fig. 4g), and these ratios fall within the range of elevated values reported from other YDB sites^45^. The background values of Pt in Pilauco sediments are higher than those for all other Pt-rich YDB sites^45^, probably due to the influence of Pt-rich basalts from nearby Andean volcanoes. In the YDB layer, however, the Pt/Pd and Au/Pt ratios are significantly higher than those of the local basalt, strongly suggesting an influx of nonlocal Pt, Pd, and Au ~12,800 years ago superimposed on higher-than-normal background concentrations of volcanic Pt.

The Pilauco results for Pt and Pd are similar to those reported by Moore *et al*.^45^ for 13 YDB sites across North America. Those workers interpreted the high concentrations of Pt and Pd as resulting from the influx of impact-related material from a nonlocal source. To possibly explain the high concentration of Pt, Pd, and Au in the YDB layer at Pilauco, we have considered the following four alternate explanations:

#### Volcanic

Are the enrichments volcanic in origin, especially since Andean basalt at Pilauco already contains high concentrations of Pt? This explanation is unlikely, however, because the Pt/Pd and Au/Pt ratios in the YDB layer are substantially different from those in the layers above and below it. This most likely indicates that the enrichment peaks are not concentrations of volcanic PGEs, but rather, resulted from the influx of Pt, Pd, and Au from some other source than the local Andean volcanoes.

#### Lag deposit

Are the enrichments in Pt, Pd, and Au possibly represent a lag deposit, formed from aeolian and/or fluvial action? This explanation is unlikely, however, because typical lag deposits do not contain high-temperature Fe- and Cr-rich spherules^1^.

#### Cosmic flux of micrometeorites

Did slow sedimentation rates cause higher concentrations of micrometeorites enriched in Pt and Pd? The sedimentation rate across the YDB layer, however, is 0.5 yr cm^−1^, which is among the fastest in the record thus eliminating this as a possibility.

#### Coincidence

Is the co-occurrence of Pt, Pd, and Au simply coincidental? If so, peaks in Pt and magnetic spherules should co-occur at random in non-YDB layers. However, no such synchronous, co-occurrence has ever been found outside of the YDB layer at Pilauco or at any other YDB site worldwide or in any other non-YDB stratigraphic layer.

#### Cosmic impact

Finally, the PGE enrichment could have resulted from the influx of nonlocal, impact-related Pt, Pd, and Au, all of which are enriched above local background levels. This is the only explanation consistent with the evidence from known cosmic impact sites and is favored for the Pilauco YDB layer. Although this evidence is not conclusive of a YDB impact event, the age for the YDB layer at Pilauco is coeval with ages for those layers found on other continents, supporting the YDB cosmic impact hypothesis.

### Section 5: Biomass Burning

**Figure 13:**
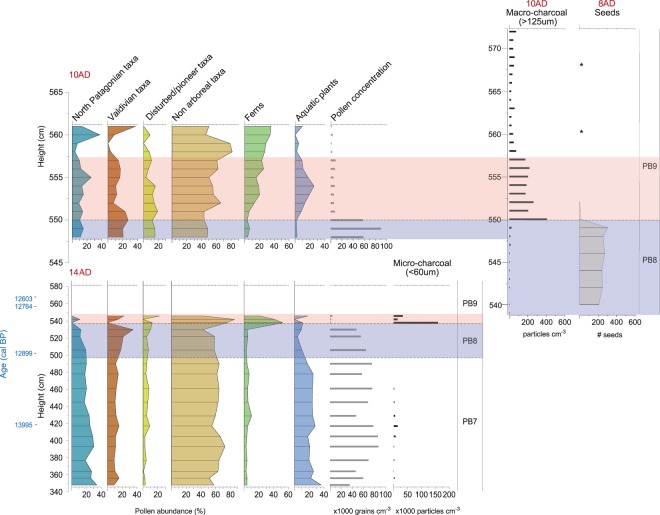
Records of pollen, seeds, and charcoal. (**a**) Pollen from grid 10AD, (**b**) pollen and charcoal from grid 14AD, (**c**) charcoal record from grid 10AD, (**d**) seed record collected in grid 8AD. Blue bands represent pre-YDB sediments (PB-8) and light red bands are post-YDB (PB-9) sediments. Non-arboreal taxa made up most of the local vegetation (40% to 85%). Below the YDB layer, North Patagonian taxa were dominant reflecting cooler, wetter climatic conditions. Coincident with the YDB, the North Patagonian forest elements were replaced by those of the Valdivian rainforest, marking a major shift to somewhat drier, warmer climate. Disturbed/pioneer taxa and ferns also show a dramatic increase at the beginning of the YDB layer associated with major fire regime.

A history of biomass burning at Pilauco was first determined by Abarzúa and Lobos^32^ from the quantitative analysis of micro-charcoal particles (<150 um) collected in grid 14AD. Charcoal is almost absent in the interval from ~16,000 to 12,800 cal BP, indicative of low fire activity in a pre-YDB interval that was marked by a cold, relatively wet climate as determined by pollen assemblage analysis. Then, beginning at ~12,800 cal BP and continuing for several hundred years, there was a major increase in charcoal abundance that indicates an anomalous episode of biomass burning (Fig. 13).

To further investigate the biomass-burning history at Pilauco, we used macroscopic charcoal concentrations (>125 um) from grid 10AD (Fig. 4l). This analysis showed low charcoal concentrations in the upper part of unit PB-8 (Fig. 4k), which pre-dates the YDB layer. Above the YDB layer, there are peak concentrations of charcoal at the base of unit PB-9, immediately following the ~12,800-year-old stratum, which is also marked by high concentrations of Pt and high-temperature impact-related spherules. Following this major peak, charcoal concentrations decline, but values remain higher than in the underlying unit PB-8. This charcoal peak is the highest in the sequence analysed and indicates an anomalously large biomass-burning episode beginning near the onset of YD climate change at ~12,800 cal BP. This biomass-burning episode appears to have contributed to significant vegetational shifts in the temperate rainforest of the Chilean Lake District^66^.

Marlon *et al*.^19^ argued against a peak in biomass burning at the YD onset across North America, but their conclusion was challenged by Wolbach *et al*.^3^, who analyzed 152 YDB charcoal records from four continents, including 28 lake sequences from nine countries across South America (five lakes are in Chile). Wolbach *et al*.^2^ argued that an anomalous episode of biomass burning occurred that is also supported by unusually large concentrations of ammonium, nitrate, formate, oxalate, and acetate in three high-resolution Greenland ice cores^67–71^. This biomass-burning peak is one of the highest of the last ~120,000 years and began precisely at the YD onset. In addition, these biomass-burning proxies are coeval with a large impact-related abundance peak in Pt^26^. In support of an association between increased biomass burning and the YDB event, Wolbach *et al*.^3^ reported in their Appendix Table A2 that the peak concentrations of YDB spherules, meltglass, and nanodiamonds are coeval with YDB biomass-burning proxies on three continents, including charcoal (23 of 32 terrestrial sites; 4 of 4 lakes), aciniform carbon/soot (7 of 14 terrestrial sites; 0 of 1 lakes), and wildfire-related carbon spherules (22 of 32 terrestrial sites; 2 of 4 lakes). Moreover, the Bayesian-modeled ages for these sites overlap the proposed age range of the YDB event between 12,835 and 12,735 cal BP.^35^ Furthermore, Wolbach *et al*.^2,3^ argued that abundant soot and combustion chemicals from such widespread biomass burning might have initiated an “impact winter,” as proposed for the K-T impact event^72,73^, although disputed by some^74^. At the YDB, this, in turn, is proposed to have contributed to YD climate change and to megafaunal extinctions across large parts of the planet. Using the evidence presented by Wolbach *et al*.^2,3^ as a guide, we observe that the YDB peak in biomass burning at Pilauco is consistent with subsequent post-YDB-impact environmental perturbations, but of course, does not prove by itself that an impact occurred. The Pilauco record is consistent with regional wildfire activity, indicating that anomalous YDB-age biomass burning reached into high latitudes of the Southern Hemisphere.

### Section 6: Plant remains and pollen

#### Fruits, Seeds, and Leaf Cuticle Record

We recovered and analysed ~4,800 carpological (reproductive) structures (e.g., fruits, seeds, and capsules) from grid 8AD between 572 and 524 cm, allowing us to infer the local pre- and post-YD vegetational history (Figs 4i and 13; Supplementary Table S10). Samples from unit PB-8 below the YDB layer contain consistently abundant carpological structures with up to 573 specimens per sample (mean 370 ± 156 SD). Leaf cuticles of evergreen tree species are common below the YDB, as represented by two pioneering trees from the Proteaceae family (*Lomatia hirsuta* and *Embothrium coccineum*) and *Amomyrtus meli*, a tree from the Myrtaceae family that requires wet soil for proper growth. Additionally, other pioneering evergreen tree species such as *Aristotelia chilensis*, *Maytenus boaria*, and the endemic conifer, *Prumnopitys andina*, are represented in the seeds record from Pilauco, together with the herbaceous species *Montia fontana* and *Gunnera tinctoria* and several varieties from the Ranunculaceae, Cyperaceae, and Plantaginaceae families.

The seeds and cuticles from nearly all of these pre-YDB plants essentially disappear from the record after the sharply defined PB-8/PB-9 boundary (Fig. 13), representing both a major decrease in abundance and diversity of plant materials and a pronounced shift in the taxonomic composition of plant assemblages. Carpological structures are ~7× less abundant above the YDB layer and remain low throughout unit PB-9, ranging from 0 to 93 specimens per sample (mean 30 ± 34 SD).

To investigate whether the decline in abundance of carpological structures reflects a decline in the local vegetation or whether the absence of plant remains is an artifact of the sampling methodology, we used a negative binomial generalized linear model (GLM) regression (see Methods). Results from the GLM analysis indicate that the major, abrupt drop in seed abundance between units PB-8 and PB-9 is statistically significant, and hence, is unlikely to have resulted from taphonomic factors such as variable transport, preservation, deposition, and/or sampling protocol^75,76^ (Supplementary Table S11).

#### Pollen Record

Similar to the carpological record, pollen concentrations remain consistently high up to the YDB layer, after which there is a dramatic and immediate decrease in abundance and diversity (species richness and Shannon index), along with a significant shift in taxonomic composition (vegetation turnover index) (Figs 4j and 13; Supplementary Table S12). A summary of the vegetation history and environmental/climatic interpretations for Pilauco and other regional sites is presented in Supplementary Table S13.

As with the fossil seeds, pollen assemblages below the YDB layer are diverse and abundant with the taxonomic composition indicating moderately dense, mostly non-arboreal vegetation dominated by Poaceae (grasses). This average assemblage is represented by a wide variety of North Patagonian taxa that decline after the YDB (Post-Pre YDB Ratio <1), including Myrtaceae (trees, 14.8%), *Nothofagus dombeyi*-type (trees, 2.3%), *Podocarpus nubigena* (conifer, 0.3%), *Pilgerodendron/Fitzroya* (conifers, 1.6%), *Tepualia stipularis* (tree, 1.1%), and *Raukaua latervirens* (vine, 1.8%). This pollen zone also includes other important plants: *Maytenus* sp. (tree, 10.8%), Cyperaceae (herbs, 2.5%), and the aquatic fern *Isoetes sp*. (0.6%). (Fig. 13; Supplementary Table S12). This assemblage is made up of mostly hygrophilous (i.e., preferring wetlands and moist environments), cold-resistant plants and trees characteristic of the North Patagonian forest, marked by cooler temperatures and relatively high rainfall/humidity^32,77–83^.

At the YDB layer (PB-8/PB-9 boundary), the percentage of non-arboreal vegetation increased sharply from ~45% to ~85% of pollen (grid 14AD; Fig. 13), representing the largest change exhibited in the pollen record from Pilauco. Many of the previously dominant taxa characteristic of the North Patagonian forest disappeared or decreased from pre-YDB levels (lower Shannon index and species richness) with a remarkable shift in vegetation turnover index from 0 to 0.4 (Fig. 13; Supplementary Table S12).

The average assemblage that increase after the YDB (post-YDB to pre-YDB Ratio >1) include the dominant taxon Poaceae from 31% to 36.7%, *Aristotelia chilensis* (tree, 8.6%), *Saxegothaea conspicua* (conifer, 5.4%), *Prumnopitys andina* (conifer, 2.5%), *Weinmannia trichosperma* (tree, 4%), *Aextoxicon punctatum* (tree, 3.6%), *Drimys winteri* (tree, 0.93%), *Eucryphia cordifolia* (tree, 0.9%), Asteraceae (herbs, 8.4%), aquatic taxa (16.4%) and ferns (17.5%) (Fig. 13; Supplementary Table S12). This post-YDB assemblage is consistently associated with an abundance of macroscopic and microscopic charcoal that indicates a general increase in wildfire frequency.

Although the carpological and pollen record at Pilauco spans only a few hundred years, numerous regional studies^66,80–82,84^ provide a solid context for the climatic interpretation of this major shift in vegetation. Despite pollen concentrations that decrease dramatically after the YDB (10× from 68,811 to 6,372 grains/cc), pollen is sufficiently abundant and diverse in the post-YDB sequence for the interpretation of broad environmental trends, especially when evaluated in the context of previous regional records. At the YD onset, drier conditions are indicated by the disappearance of aquatic ferns (*Isoetes* sp.; grids 10AD and 14AD), following their persistent presence throughout the pre-YDB sequence. These changes were also accompanied by increased biomass burning fostered by the onset of a marked seasonality of rainfall and warmer conditions. Furthermore, beginning at the YDB layer the vegetation records show a distinct upward increase (from 5 to 20%) of pioneering/colonizing taxa indicative of more disturbed open habitats (Fig. 13)^32,77–83^. These taxa appear to have colonized the area in response to the abrupt onset of alternating seasonal drying and precipitation (humidity) that also promoted an increase in biomass burning. All of these factors indicate that the post-YDB vegetation at Pilauco is closely affiliated with the more northern Valdivian rainforest that favors seasonally drier and warmer conditions. As such, the vegetation history at Pilauco records a sudden shift at the YDB from cooler, wetter conditions characteristic of the North Patagonian forest to seasonally drier and warmer conditions of the Valdivian rainforest. Coinciding with the termination of the Antarctic Cold Reversal, this shift in vegetation at the YDB continued for at least 100 years, suggesting persistent post-YDB climate change and a disturbed landscape.

The plant and charcoal records from Pilauco are complex, but crucial for understanding regional climate change, and so, we summarize our findings here. Although most investigations in the Lake District, Chiloe Island, and Pilauco show variability among sites before, during, and after the YD Chronozone, essentially the same forest/climate succession and environmental disruption is evident among all the records across the YD boundary (e.g. Moreno *et al*.^84^, and see Supplementary Information, S2 “Background: Paleoclimate and Biota”, and Supplementary Table S13 for a list of investigations in the area). More research is necessary to confirm the presence of impact-related evidence at other sites in southern Chile.

### Section 7: Climate change at the YDB

The high-resolution climatic record at Pilauco allows us to test the correlation of local climate at ~12,800 cal BP with similar changes across a much wider area. The result is a novel synthesis showing the onset of abrupt, coeval, pan-hemispheric climatic change. This is suggestive, but not conclusive of causation by a cosmic impact event at the YD onset, which may be represented by the recently discovered Hiawatha Crater in Greenland^25^.

In southern Chile located between 40° and 44°S, colder-to-warmer and wetter-to-drier climate change at the beginning of the YD at ~12.8 ka, as discussed in the section above, has previously been documented for several sediment sections^66,80,81,84^. This change is associated with the termination of the Antarctic Cold Reversal episode, anti-phased with the shift in the Northern Hemisphere from the warm Bølling-Allerød to the colder YD. The Pilauco record shows that this climate shift occurred very rapidly within just a few years or less, based on sedimentary deposition rates. It appears that the abrupt changes in southern Chilean vegetation (cool/wet North Patagonia forest to warmer/drier Valdivian rainforest), marking the YD onset and the termination of the Antarctic Cold Reversal, were caused by the abrupt southward shift of the southern westerly wind belt towards the Southern Ocean and Antarctica^66,80,81,84^.

**Figure 14:**
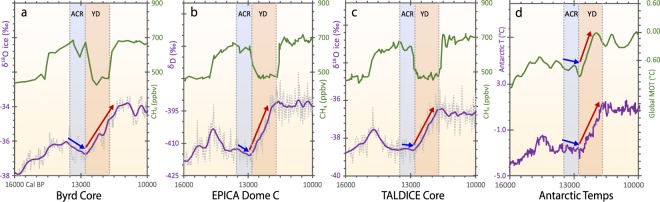
Antarctic climate record exhibiting warming trend at ~12,800 cal BP. Changes in methane (CH_4_) concentrations are used to identify and correlate climate change boundaries representing the Antarctic Cold Reversal and the Younger Dryas. Climatic history is reflected by changes in δ^18^O and δD values, which are proxies for temperature, with lower values indicating cooler climate. (**a**) Byrd core in West Antarctica; (**b**) EPICA Dome C near the center of East Antarctica; (**c**) TALDICE core from Talos Dome in southern East Antarctica; (**d**) green upper curve represents global mean ocean temperature from WAIS divide near Byrd in West Antarctica. Purple lower curve represents atmospheric temperatures reconstructed from CO_2_ record at EPICA Dome C^140^. Blue vertical bar represents cooler climate during the Antarctic Cold Reversal, anti-phased with the warm Bølling-Allerød episode in the Northern Hemisphere. Orange vertical bar represents warmer climate in Antarctic coeval with Younger Dryas cooling in the Northern Hemisphere. Blue and red arrows indicate colder or warmer temperature changes, respectively, across the Antarctic Cold Reversal and the Younger Dryas.

The changes in Southern Chilean climate at the YD onset are broadly similar to other changes described at high southern latitudes. Evidence includes well-dated δ^18^O and δD proxy climate records in Antarctic ice cores (Byrd Station^85^; EPICA Dome C^86,87^; and TALDICE^88,89^), as well as Vostok^90^; EPICA Dome ML^91,92^; and Taylor Dome^93^. This change is confirmed by a reconstruction of Antarctic atmospheric temperatures at EPICA Dome C^86,87^. As at Pilauco, these records exhibit an abrupt cold-to-warm climate shift at ~12.8 ka, representing the end of the Antarctic Cold Reversal (Fig. 14).

The cold-to-warm climatic shift recorded in the YDB layer in southern Chile sharply contrasts with the warm-to-cold shift that sometimes occurred at the YD onset in the Northern Hemisphere^94–96^. This differing climatic response between the Southern and Northern Hemispheres at the YD onset appears to have been prompted by two synergistic processes^97^ associated with anti-phased, bipolar, climate shift (meridional seesaw effect)^94–96^, resulting in immediate corresponding climate responses via the atmosphere, rather than slower responses through oceanic processes. Possible contributing mechanisms include dramatic outburst flooding from destabilized ice sheets and proglacial lakes at the YD onset in the Northern Hemisphere^4,98–101^ that injected massive quantities of meltwater and ice into the adjacent Arctic and North Atlantic Oceans, thus reducing sea surface salinities and expanding sea-ice across the Arctic and high latitudes of the North Atlantic. This, in turn, strongly reduced North Atlantic meridional overturning circulation and curtailed production of North Atlantic Intermediate Waters, further strengthening cooling and sea-ice expansion in the Northern Hemisphere. This major change in circulation in the Northern Hemisphere helped to stimulate Antarctic deep-water production in the Southern Hemisphere and its northward expansion in the world’s oceans. The abrupt YD cooling episode in the Northern Hemisphere, in turn, prompted changes in global atmospheric telecommunication represented by a sequence of southward shifts in the zonal wind systems in response to the changes in sea-surface temperature gradients.

The Inter Tropical Convergence Zone (ITCZ) is a belt of low pressure which circles the Earth, generally where the trade winds of the Northern and Southern Hemispheres come together near the equator. During climatic transitions of the late Quaternary, tight coupling is known to have existed between the southern margin of the ITCZ and the southern westerly winds^97,102,103^. At the onset of the YD, the ITCZ was pushed southwards farther into the Southern Hemisphere, and this, in turn, resulted in extreme southward displacement of the southern westerlies^97^, as reflected in southern Chilean sequences, including Pilauco. This is an important process, because changes in the latitudinal position and strength of the southern westerlies strongly affect ocean circulation in the Southern Hemisphere and strengthen warming south of 35°S^97^, including the Southern Ocean and Antarctica. At the onset of the YD, this was reflected by a reduction in sea ice extent and increased wind-driven upwelling and ocean overturning^97^. In turn, this led to the increased release of carbon from the deep ocean, to outgassing of CO_2_ into the atmosphere due to Southern Ocean upwelling, and to a rise in atmospheric pCO_2_. It is also possible that these changes led to increased production of Antarctic Bottom Water at the YD onset, coeval with a major decrease in North Atlantic meridional overturning circulation (the ocean conveyor).

The YD onset is also dramatically and enigmatically marked by an abrupt rise in mean ocean temperatures in both hemispheres that continued for ~700 years^104^ (Fig. 14d). This unprecedented ocean warming episode required massive oceanic energy uptake of ~10^21^ joules per year, representing the fastest and strongest global ocean warming episode of at least the last 22,000 years, the limit of the record investigated^104^ and surpassing modern multi-decadal warming trends recorded from 1971 to 2005^104^. The rapidity and magnitude of this ocean warming episode during the early and middle YD is difficult to explain^104^, but may be related to the impact into the Greenland ice sheet that created the Hiawatha crater^25^, possibly at ~12,800 cal BP. This warming trend is just one of multiple extreme characteristics marking the YD episode. It is possible that the warming at high southern latitudes, resulting from the southward shift of the southern westerly winds and the South Pacific Tropical High, contributed to the major increase in heat transfer necessary to significantly warm the deep Southern Ocean, which in turn, would have reduced sea ice extent, as well as stimulated ocean turnover and production of deep waters. The loci of Antarctic deep water production likely shifted to the north in the warmer, ice-free, open Southern Ocean, where the production of warmer deep waters would have been dominated by open-ocean convection processes^105^. This shift would have enhanced heat transfer to the deep Southern Ocean, especially in absence of North Atlantic Deep Water formation, and may help explain the major, highly unusual rapid increase in mean ocean water temperatures for 700 years during the early and middle YD^104^.

The evidence suggests that a seemingly enigmatic chain of interconnected oceanic and atmospheric circulation changes caused the YD climatic episode to be expressed simultaneously in both the Northern and Southern Hemispheres. These changes were marked by anomalous timing and by the character and magnitude of changes in continental meltwater plumbing, accompanied by major shifts in atmospheric and oceanic circulation. We posit that the YDB cosmic impact event, possibly resulting in the Hiawatha Crater, triggered these processes and that they appear less enigmatic in the context of a cosmic impact triggering mechanism.

The boundary between the warm Bølling-Allerød climatic episode (14,500 to 12,800 cal BP) and the subsequent cold YD episode in the Northern Hemisphere precisely correlates with anti-phased climate change in the Southern Hemisphere. This boundary in the Southern Hemisphere, as well documented at Pilauco, represents a useful isochronous datum for intercontinental correlation and dating of deglacial sequences^35^. The YDB datum layer, regardless of its origin, allows precise interhemispheric correlation of climate change at ~12,800 cal BP.

### Section 8: Human-megafaunal interactions at Pilauco

**Figure 15:**
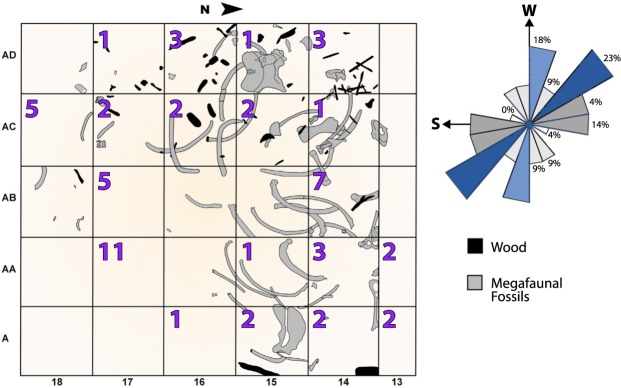
Grid map of megafaunal (proboscidean) bone and human lithic distributions. Bones (gray) mapped during the excavation are oriented to the SW-NE (23%) and SSW-NNE. Orientation of the proboscidean bones suggests the animals died *in situ*. Carnivores were possibly responsible for alteration and/or removal of some bones. Purple numbers represent the quantity of human lithics found in each grid. There is no current evidence that humans modified the bones, although the presence of numerous closely associated lithics suggests significant human interaction with the extinct proboscidean remains.

Five of the 40 1-m^2^ excavated grids produced 140 human lithics, including unifacial artifacts, flakes, and small pieces of debitage^34^, the latter accounting for the highest percentage (75%) with cores and edge-trimmed artifacts representing 12% and 13% of the total, respectively (Fig. 15). Most artifacts and flakes were found in unit PB-7 between a height of 361 and 424 cm in grids adjacent to or including bones of gomphotheres (elephant-like proboscideans) (cf. *Notiomastodon platensis*). Obsidian and dacitic glass micro-debitage are also found in these same areas. Concentrations of aphanitic and vitreous lithic materials also coincide with areas containing abundant ribs and long bones of gomphotheres^27,34^. Investigators also found a human footprint associated with the megafaunal remains in unit PB-7^30^ and a manually perforated seed that was most likely used as an ornament^27^. For more on human-related lithics, see Supplementary Information, S4.

Lithics produced by the first human settlers at Pilauco are only found in close association with megafaunal remains. This observed connection suggests that humans were exploiting extinct megafauna through scavenging and/or hunting. No human artifacts were found after the extinction of the megafauna at the YDB layer and PB-8/PB-9 boundary. This absence of remains suggests that humans either abandoned the area after the megafaunal extinctions and/or experienced a regional population decline and/or reorganization, similar to that proposed for North America^106^.

### Section 9: Spores of *Sporormiella* spp. (dung fungi)

The Pilauco site is best known for its late Pleistocene megafaunal fossil assemblage that exhibits extinctions of a number of these taxa^27^. To enhance our biostratigraphic investigation of the Pilauco megafauna and their extinctions, we quantified the vertical distribution in these sediments of coprophilous fungi spores (e.g., *Sporormiella* spp., *Sordaria* spp., and *Posospora* spp.). These spores are commonly found in the faeces of livestock, such as cows and horses, as well as in that of extinct megaherbivores such as mammoths, ground sloths, and horses^13,107^. Experimental research with modern bison populations demonstrates that *Sporormiella* spp. spore concentrations are a reliable local-scale proxy of megaherbivore population sizes^108^ and are widely used in studies of extinct megafauna^109,110^. The correlation between extinct megaherbivore dung and *Sporormiella* spp. spores was established after the discovery of a layer of fossilized mammoth dung in Bechan Cave, Utah^109,110^. Variations in abundance of these coprophilous fungi in Pleistocene- and Holocene-aged sediments are considered to be directly proportional to the local abundance of megaherbivores, and thus to provide a reliable indicator of the presence, relative abundance, decline, and extinction of megafauna populations on several continents^108–115^.

**Figure 16:**
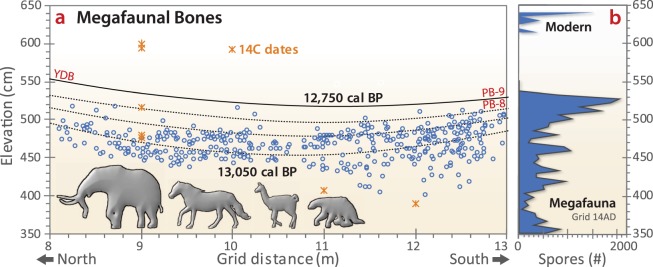
Stratigraphic distribution of megafaunal bones and *Sporormiella* spp. dung fungi spores. (**a**) Fifty-year-spaced isochrons for several grids across the excavation site are based on a Bayesian age-height model. The ~12,750-year isochron represents the YDB layer at the boundary between units PB-8 and PB-9. Extinct megafaunal remains were found below that isochron, with only non-extinct taxa found above. Data on stratigraphic locations of megafaunal remains are from Recabarren *et al*.^121^; Pino *et al*.^27^; and Labarca *et al*.^29^. (**b**) Biostratigraphy of *Sporormiella* spp. spores: A major peak in *Sporormiella* spp. concentration is shown at the YDB layer and immediately below after which *Sporormiella* spp. abruptly disappears from the record for hundreds of years. *Sporormiella* spp. in sediments from the uppermost part of the section are inferred to be associated with extant megafauna such as the guanaco (Camelidae) and huemul (Cervidae).

At Pilauco, the sediment layers below the YDB (units PB-7 and PB-8) contain persistent but variable concentrations of *Sporormiella* spp. spores in association with common megafaunal bones (Fig. 16a). Maximum concentrations of *Sporormiella* spp. spores were found in the lower part of unit PB-7 (1,540 spores cm^−3^) at ~16,500 cal BP (Fig. 16b) and in the upper part of unit PB-8 (1,800 spores cm^−3^), which is immediately below the YDB layer (PB-8/PB-9 boundary). Above this boundary, *Sporormiella* spp. spores abruptly disappear from the record, indicating a major megafaunal population decline or extinction. Spores reappear in much lower numbers hundreds of years later near the top of unit PB-9 most likely caused by an inferred influx of modern herbivorous fauna such as *Lama guanicoe*, *Hippocamelus bisulcus*, and *Pudu puda*.

The large peak of *Sporormiella* spp. spores, followed by their abrupt disappearance, suggests that extinct megafaunal populations were large and robust just prior to the YD onset, after which they abruptly disappeared. This suggests a possible causal connection between the megafaunal extinction and the cosmic-impact event. Records of dung fungi from North America appear more equivocal (e.g., Gill *et al*.^108^ and references therein) with limited regional megafaunal extirpations and possibly even extinctions underway prior to the YD onset.

### Section 10: Megafaunal Extinctions

#### Megafaunal extinction record in southern South America

Prior to the Pleistocene, several confirmed impact events are known to have been associated with megafaunal extinctions across South America (Supplementary Information, S5), although it should be noted that not all known impacts caused extinctions. For the late Pleistocene extinctions, the extent and magnitude are not particularly well known but are the subject of continuing investigations^116–119^. Altogether, South America lost more Pleistocene genera (~52) and species (~66) than North America, Europe, or Asia and it is also known that the majority of these extinctions occurred during a relatively brief interval close in age to the Allerød/YD boundary. Villavicencio^119^, for example, suggested that >80% of South American mammals over 44 kg became extinct close to the onset of the YD, although several studies have reported that members of some extinct species lingered on for several thousand years before finally becoming extinct^17,117,118^. For the southern Patagonian region, radiocarbon ages of five megafaunal taxa, with relatively continuous biostratigraphic records, suggest an end-extinction close to ~12,500 cal BP, which is a few hundred years younger than the YD onset^120^. At Pilauco, we investigated how the extinction record compares with that for the rest of southern South America, as well as that for North America.

#### Megafaunal extinctions at Pilauco

Pino *et al*.^27^ reported 112 bones, representing a minimum of eight extinct megafaunal taxa (Figs 15 and 16a), including the gomphothere (cf. *Notiomastodon platensis*), American horse (*Equus* (*Amerhippus*) *andium*), one sloth (*Xenarthra* indet.), and one camelid (cf. *Hemiauchenia paradoxa*)^27,29,31,33,121^. Most of these megafaunal bones were found in unit PB-7, while the overlying unit PB-8, immediately below the YDB layer, contains only extinct horse remains. For example, a coprolite from an extinct horse was found in unit PB-8 a few cm below the YDB layer and has a calibrated age range of 13,065–12,749 cal BP (mean age of 12,907 ± 158 cal BP; 11,004 ± 186 ^14^C years BP)^27^.

**Figure 17:**
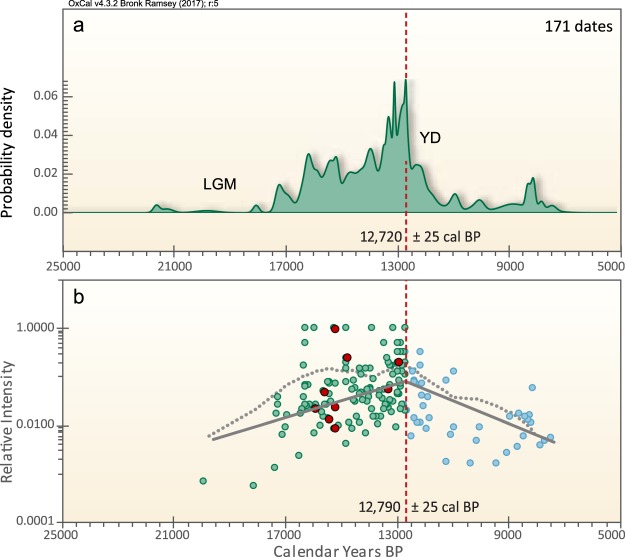
Statistical analyses of megafaunal radiocarbon dates. (**a**) Summed probability for 171 previously reported radiocarbon dates on megafaunal remains mostly from southern Chile and Argentina. The highest peak in stacked radiocarbon dates occurs at ~12,720 ± 25 cal BP, immediately below the YDB impact layer. (**b**) Spacing analysis of the same 171 radiocarbon dates, clustering at ~12,790 ± 25 cal BP. Gray trend line represents a linear regression, and the light gray dotted line represents lowess smoothing of the data. Green circles are pre-YDB in age and blue circles are post-YDB. Both trend lines indicate a general increase in numbers of radiocarbon dates from ~16,000 to ~12,800 cal BP, followed by a conspicuous decline in the number of dates, which is inferred to represent a major decline in megafaunal populations. The large peak in both plots at ~12,800 cal BP appears to be the time at which the YDB cosmic impact abruptly triggered large megafauna declines, including extinctions and extirpations of many taxa, later followed by full extinction of the remaining taxa.

Distinctly fewer megafaunal bones occur in the uppermost levels of unit PB-8 (Fig. 16a), which at face value, suggests a decline in megafauna populations during the ~100-yr interval prior to the YD onset at ~12,750 cal BP. Concentrations of *Sporormiella* spp. spores, however, show no comparable decline (Fig. 16b), but instead exhibit the highest spore concentrations in the record before disappearing at ~12,750 cal BP, as has been found at other sites across the Americas^110^. This result suggests that extinct megafauna remained continuously abundant at Pilauco right up to their local extinction, coincident with the YD onset (Fig. 17). Villavicencio *et al*.^119^ reported that 93% of the dates on megafaunal remains (n = 62 of 67) are either older than or fall within the age range of the YDB impact event at 95% certainty (12,835 to 12,735 cal BP^35^). These extinct taxa include ground sloth (Mylodontidae), American horse (Equidae), saber-toothed cat (Felidae), jaguar (Felidae), bear (Ursidae), fox (Canidae), and several camelids.

#### Megafaunal extinctions across southern South America

We analyzed 171 previously reported radiocarbon dates of extinct late Pleistocene megafaunal fossils from across southern Chile and Argentina using two statistical techniques, summed probability analysis^106^ and spacing analysis^122^. Both techniques assume that the number of radiocarbon dates increases or decreases in direct correlation with increases or decreases in size of extinct megafaunal populations. This assumption is diminished due to biases in sample collection, sample size, preservation of megafaunal remains, and variability in the accuracy and precision of the radiocarbon dates^106^. Despite the potential biases and inaccuracies, these two statistical methods are generally accepted as providing accurate first-order approximations of long-term changes in megafaunal population sizes^106,122^. To avoid any bias due to the use of different calibration curves, we used original radiocarbon dates and calibrated them using the same southern hemispheric calibration curve, SHIntCal13, within the OxCal program version 4.3.2.

The first method, summed probability analysis, is a Bayesian analytical technique that involves calibrating megafaunal radiocarbon dates and stacking the probability ranges using the SUM algorithm in the OxCal program. Major peaks and troughs in the trends are inferred to be directly proportional to changes in megafaunal population densities^106^. The advantage of this technique is that it utilizes the entire range of uncertainties for the calibrated dates. The 171 dates from Chile and Argentina are from remains of the extinct armadillo (superorder: Xenarthra; n = 11), horse (family: Equidae; n = 27), jaguar (family: Felidae; n = 6), guanaco (family: Camelidae; n = 15), mostly mastodon-like gomphothere (order: Proboscidea: n = 11), a rhinoceros-like species (suborder: Toxodonta; n = 4), saber toothed cat (genera: Smilodon; n = 5), short-face bear (family: Ursidae; n = 1), and sloth (families: mostly Mylodontidae and Megatheriidae; n = 91).

Our results suggest that population sizes of the extinct megafauna broadly increased after ~18 ka during the Last Glacial Maximum (LGM) and continued to increase throughout the Antarctic Cold Reversal, even though temperatures were colder and seemingly less hospitable. Both the LGM and the Antarctic Cold Reversal were as cold or colder than the YD episode in the Northern Hemisphere (Fig. 17a). Megafauna populations continued with a large, conspicuous increase until ~12,720 ± 25 cal BP, when population levels abruptly plummeted by ~60% within less than a few centuries, marking the largest drop in ~22,000 years, the limit of the record. The age of the main population peak closely matches that for South American populations reported by Surovell *et al*.^122^, and the population decline overlaps with the YD onset at ~12,800 cal BP^35^. Following the onset of YD climate change, the reported ages of extinct megafauna indicate that some taxa persisted at very low population levels until becoming extinct during the YD interval and early Holocene.

We also used spacing analysis for the same 171 radiocarbon dates, following the analytical method of Surovell *et al*.^122^. This second technique compiles the statistically inverted gaps or time lags between consecutive dates and infers the sizes of extinct megafaunal populations based on the assumption that wider gaps between successive dates indicate smaller populations and vice versa. The disadvantage of this approach is that it uses only mean calibrated ages without considering their inherent uncertainties. Spacing analysis shows that megafaunal populations peaked at ~12,810 to 12,780 cal BP (Fig. 17b) followed by a large abrupt decline in their populations beginning at or near the YD onset. Subsequently, small populations of some taxa persisted, also to decline and become extinct during the YD and early Holocene. These data closely match the results of the summed probability analysis, and overlap with the age range of 12,835 to 12,735 cal BP for the proposed YDB impact event.

The megafaunal extinctions were clustered at the end of the Pleistocene across several continents, although the overall pattern was complex^17,112,122^. At Pilauco, the record shows that the local extinctions coincide with the YDB layer. However, elsewhere across southern South America, there are questions whether some megafauna became extinct prior to the YD onset and whether others became extinct later in the early Holocene. In any event, the evidence at Pilauco is consistent with the hypothesis that the proposed YDB impact event contributed to the South American megafaunal extinctions.

### Section 11: Impact scenario

The YDB impact event is argued to be the product of an astronomical environment that is discussed in detail in Wolbach *et al*.^2^ Summarizing that discussion, astronomical discoveries over the last few decades show that mass distribution of comets appears to be disproportionately large for bodies with diameters near and up to 300 km, representing a greater hazard for Earth impacts. A 250-km comet with typical density of 0.5 g/cm³ has 1000 times the mass of the entire current near-Earth asteroid system, so that Earth impacts from cometary material are more likely than from a similar-sized asteroid^2^. These large bodies drift into the near-Earth environment quite frequently in geological timescales, and in fact, the broad remains of two such bodies are present in the inner Solar System today. One of them, the Taurid Complex, is composed of debris from an ~100-km-wide comet that arrived at least 20,000 to 30,000 years ago from the centaur system of large comets and then, further disintegrated hierarchically in a short-period, Earth-crossing orbit^123,124^. There is a reasonable probability of one or more encounters within the last 13,000 years with debris swarms from the Taurid Complex or other large fragmented comets, and such an encounter would be hemispheric in scope, lasting for only a few hours. The resulting debris field would be a mixture of dust and larger fragments, potentially equivalent to the impact of ~1000 to 10,000 destructive airbursts, such as occurred in Tunguska, Siberia in 1908^125^. If such an event occurred at the YD onset, larger objects in the debris swarm could have created craters on land, struck the world’s ice sheets, and/or impacted the world’s oceans, creating severe biotic and climatic disturbances^126^.

### Section 12: Conclusions

The main objective of this study was to test the YDB impact hypothesis by analyzing a wide range of data from the Pilauco site in southern Chile. The following conclusions show that our data and interpretations are consistent with the YDB impact hypothesis and we found no evidence that refutes the hypothesis.

1. At Pilauco, ~12,800-year-old peaks in high-temperature Pt-rich and native-Fe spherules are comparable to similar impact-related evidence found at more than 50 YDB sites in North America, Europe, and western Asia. It appears that the YDB layer at Pilauco is coeval with similar layers found at these sites on several continents and is also possibly related to the proposed YDB impact event.
2. Identification of the YDB layer at Pilauco greatly expands the proposed YDB proxy field ~6,000 km farther south of the closest well-studied YDB site in Venezuela, and ~12,000 km south of the northernmost YDB site in Canada, a distance equaling ~30% of Earth’s circumference.
3. Cr-rich spherules are found in the YDB layer at Pilauco, but not found at the ~50 other sites on four continents, suggesting that one or more local impacts/airbursts occurred in the Cr-rich basaltic terrain circa Pilauco.
4. Unusually high Pt/Pd and Au/Pt ratios suggest the influx of non-local PGE-rich material. The presence of a significant Pt abundance peak in the YDB layer at Pilauco supports the proposition that the proposed YDB impact event was large enough to have distributed impact-related materials across both the Northern and Southern Hemispheres.
5. The Pilauco charcoal record shows that the largest episode of biomass burning in the sedimentary sequence investigated was coeval with the widespread biomass-burning event at the YD onset, as identified in >150 ice, lake, and terrestrial records from four continents, including lakes in South America.
6. Pollen, cuticles, and seeds records from Pilauco exhibit a large decline in abundance and diversity, and a major change in taxonomic composition of plants at the YD onset. These changes indicate there was significant environmental disruption at Pilauco, associated with abrupt climate change. Although the change in the abundance/diversity of plant taxa and fire activity recorded at the Pilauco site cannot be solely attributed to an extra-terrestrial impact, all the independent lines of evidence presented in our investigation (spherules, trace elements, fossil plants-animals, etc.) suggest that these changes may have been driven by an extra-terrestrial event.
7. The YDB layer at Pilauco coincides precisely with the abrupt termination of cooler climate of the Antarctic Cold Reversal across southern South America and Antarctica, immediately followed by the onset of warmer conditions. Through the seesaw effect, this climatic change is anti-phased via atmospheric processes with Northern Hemisphere cooling at the YD onset.
8. Human artifacts were found at Pilauco only below the YDB layer, suggesting a local population reorganization/decline. They were found only in association with extinct megafauna bones.
9. At Pilauco, no bones or remains of extinct megafauna were found above the YDB proxy layer. Similarly, the record of *Sporormiella* spp. spores indicates that the local megafauna extinction was coeval with deposition of high concentrations of impact spherules and Pt, suggesting a possible causal connection to the YDB impact event.

In summary, evidence has been found in the Pilauco section that is similar to that found at >50 YDB sites on four continents. This is the first time that extensive YDB evidence has been found at high latitudes in the Southern Hemisphere. The evidence reported in this study appears consistent with the proposed effects of a YDB cosmic impact event that affected both the Northern and Southern Hemispheres.

### Section 12: Methods

#### Stratigraphic sampling

A 280-cm-long, 5-cm-wide column was collected from grid 14AD that included units PB-7, PB-8, and PB-9. Later, in 2014, a 34-cm-wide column was collected from grid 10AC to develop a detailed stratigraphic sequence across the PB-8/PB-9 boundary. Grain-size analyses by wet sieving and loss-on-ignition (LOI) were performed at 1-cm intervals along both stratigraphic columns to quantify proportions of gravel, sand, mud, and organic fractions. Sedimentological sampling and analyses were performed at the Universidad Austral de Chile, Valdivia, by co-author M.P. with the assistance of Javiera Barria and Daniel Fritte. Subsequently, co-author M.P.L. developed the stratigraphic profile.

#### Radiocarbon dates

A total of 36 AMS radiocarbon dates were obtained for this study from bulk sediment, seeds, charcoal, wood, plant remains, bone, teeth, and coprolites (Supplementary Table S4). Samples were acquired in Chile by M.P. with the assistance of Daniel Fritte. Nine radiocarbon dates are from Pino *et al*.^27^ and 27 others were obtained for this study. Twelve dates were measured by the NSF-Arizona AMS facility, University of Arizona and 24 are from the W. M. Keck Carbon Cycle Accelerator Mass Spectrometry Laboratory, University of California, Irvine. Samples for radiocarbon dating were collected from excavation grids 6E, 7AC, 7 G, E9, 10AD, 11 H, 12 F, 14AD, 15AC, and 15AD (Supplementary Figs S1–S3). Twenty AMS radiocarbon dates were outside the interval of interest, and the remaining sixteen were used to develop an age-height model (see Methods; Fig. 3; Supplementary Table S5). These dates were calibrated within the OxCal program 4.3.2^127^, using the Southern Hemispheric calibration curve, SHIntCal13. Uncertainties were calculated at confidence intervals (CI) of 68% and 95%. The layer dating to ~12,800 cal BP was identified in grid 8AD at a height of ~550 cm above the site datum (elevation, not depth). Because stratigraphic heights varied by centimeters to decimeters in other grids, the YDB layer in all grids is correlated and normalized to 550 cm in grid 8AD, allowing stratigraphic and chronologic comparisons among the various proxies measured in the different grids.

#### Analyses of high-temperature impact spherules

Twenty-three bulk sediment samples (~1 kg each) from grid 8AD were examined across a 138-cm-thick profile that ranged in height from 651 cm at the surface to 513 cm at its deepest point. Continuous samples were taken between 572 and 524 cm, with one additional discontinuous sample each taken at the top and bottom of the section. Sample thicknesses averaged ~3 cm along the sediment profile. From 552 to 548 cm, samples were 1-cm thick. The four samples collected below and above these were 2-cm thick, and samples at the top and bottom of the profile were 4-cm thick. Samples were collected by M.P. and Daniel Fritte.

To determine the composition of spherules from Pilauco, we used scanning electron microscopy and energy dispersive x-ray spectroscopy (SEM-EDS), and ternary geochemical analyses to compare the YDB spherules from Pilauco to other known types of spherules. David Kimbel in the US extracted the magnetic fractions and Jennifer Zeldin in the US mounted candidate YDB spherules for SEM analyses. M.A.L. and V.A. performed the SEM-EDS analyses using a JEOL JSM 6010PLUS/LA at Elizabeth City State University and a Hitachi S3200N variable pressure scanning electron microscope (VPSEM) at North Carolina State University. All SEM imagery was acquired at a resolution of 2560 × 1920 pixels. Images were uniformly post-processed for contrast and brightness, if necessary, using Adobe Photoshop CC2014.

#### Quantification of platinum

We used the methodology described in Moore *et al*.^45^. Sediment samples of approximately 50 grams each were collected by M.P. and Javiera Barria and then sent for analysis by Activation Laboratories (ActLabs), Canada using their “1C-Research” package. The package includes fire-assay (FA) and inductively coupled plasma mass spectrometry (ICP-MS) to measure elemental concentrations of Pt, Pd, and Au. Prior to analysis, each sample was mixed with fire assay fluxes (borax, soda ash, silica, litharge) and silver (Ag) was added as a collector. The mixture was placed in a crucible and preheated to 850 °C, intermediately heated at 950 °C, and finished at 1060 °C for a total of 60 minutes. After the crucibles were removed from the assay furnace, the molten slag was poured into a mold leaving a lead button. The lead button was then preheated to 950 °C to recover the Ag (doré bead) mixed with the extracted Au, Pt and Pd. The Ag doré bead was digested in hot (95 °C) HNO_3 + _HCl with a special complexing agent to prevent the Au, Pd, and Pt from adsorbing onto the test tube. After cooling for 2 hours, the sample solution was analyzed for Au, Pt, and Pd using a Perkin Elmer Sciex ELAN 9000 ICP-MS. On each tray of 42 samples there were 2 method blanks, 3 sample duplicates, and 2 certified reference materials. The ICP-MS was recalibrated every 45 samples. Smaller sample splits were used for high-chromium and sulfide samples. Measurements are reported in parts per billion (ppb) with a lower limit of detection for Pt of 0.1 ppb. All control testing indicated that the analyses were accurate and replicable.

#### Analyses of plant remains

The same 138-cm-thick profile examined for the presence of impact spherules in grid 8AD was also investigated for plant remains (fruits, seeds, capsules, cuticles). Sediment samples were collected and analyzed by M.P., Daniel Fritte, and Javiera Barria. The sediment samples were wet sieved through a series of sieves (2 mm to 63 µm) for the separation of gravel, sand, and mud. Next, the fractions were oven-dried at 60 °C, dry weighed, and stored in plastic bags for further examination. The fractions from each sample were examined under a stereomicroscope at 10 × to 40 × magnification for the picking and sorting of reproductive structures (i.e., seeds, fruits and capsules; hereinafter referred to as seeds) and leaf cuticles. Plant remains were analyzed by G.A. at the Universidad Austral de Chile, in Valdivia.

Numerical analyses were performed only on the seed data because of the high fragmentation of cuticle remains and uncertainty in knowing the number of individual leaves from which the cuticle fragments were derived. We tested whether variation of our count response variable (number of seeds) for units PB-8 and PB-9 was affected by different sample sizes (thickness and/or total weight of the sample). To do so, we fitted a Poisson GLM regression, in which the dependent variable is allowed to have a non-normal distribution. This regression holds that any observed response is a linear sum of multiple underlying individual responses. Because we detected over-dispersion (greater than expected variance from the mean), a correction of the standard errors was performed using a quasi-GLM model specifying the mean and variance relationship (i.e., variance given by φ × μ, where μ is the mean and φ the dispersion parameter), following the method of Zuur *et al*.^128^. Finally, we fitted a negative binomial GLM and compared the two approaches by using the log-likelihoods of the negative binomial regression model and a Poisson regression model using the odTest function of the ‘pscl’ package in R. All analyses were conducted in R^129^.

#### Analyses of pollen

For quantification of pollen grains and spores, we analyzed 13 contiguous samples of 1 cm^3^ (548 to 561 cm height) near the PB-8/PB-9 boundary in grid 10AD following the standard palynological procedures outlined by Faegri and Iversen^130^. Pollen samples were analyzed by co-authors, A.M.A. and A.M-C. at the Universidad Austral de Chile in Valdivia. Chemical treatment included deflocculation with 10% KOH, sieving to remove large debris (150 μm), silicate dissolution with 40% hydrofluoric acid (HF), acetolysis with 10% KOH, and concentrated HF and acetolysis. The concentrates were mounted in glycerin and analyzed at 400× and 1000× with a Zeiss microscope. Taxonomical determinations were made based on published descriptions^131^, and modern pollen reference collections held at the Palynology and Environmental reconstructions laboratory, Universidad Austral de Chile. From each slide, a minimum of 300 pollen grains from terrestrial taxa were counted, and concentrations were calculated based on *Lycopodium* spores added to the preparations. Percentages of pollen abundances are based on the total sum of pollen. Pollen diagrams and cluster analysis (CONISS) were produced using the software Tilia^132,133^.

#### Analyses of charcoal

To investigate the biomass-burning history of the Pilauco section, we performed macroscopic charcoal analysis on samples from a 38-cm-long profile including the PB-8/PB-9 boundary in grid 10AD, located on the west wall of the site. We analyzed 2 cm^3^ of sediment taken at contiguous 1-cm intervals along the complete section. The samples were sieved using the methods outlined by Whitlock and Larsen^134^. The charcoal fractions (>0.125 mm) were counted in gridded Petri dishes under a stereomicroscope and expressed as charcoal concentrations (particles cm^−3^). Charcoal samples were analyzed by A.M.A. and A.M-C. at the Universidad Austral de Chile in Valdivia.

#### Analysis of *Sporormiella* spp. fungi spores

Counts of *Sporormiella* spp. spores were performed in 76 of the pollen preparations^32^ from a stratigraphic section in grid 14AD that includes units PB-7, PB-8, and PB-9. A magnification of 400× was used, and added *Lycopodium* spores were used to calculate concentrations. The determination of *Sporormiella* spp., characterised by its sigmoidal germinal opening and dark brown color, was performed using keys, photographs, and morphological descriptions (cf. refs^135–138^). Spores were analyzed by N.C-M. at the Universidad Austral de Chile in Valdivia.

#### Statistical analyses of megafaunal remains

Spacing analysis was used to investigate the gaps or time lags between consecutive calibrated radiocarbon dates. This technique assumes that mean spacings between calibrated ages are inversely proportional to the size of extinct megafaunal populations (i.e., smaller intervals mean larger populations). To avoid inaccuracies introduced by the use of multiple calibration curves, we did not use calibrated dates from the original studies. Instead, co-author A.W. recalibrated all original radiocarbon dates with the OxCal program, version 4.3.2^127^, using SHCal13, the radiocarbon calibration curve for the Southern Hemisphere. After inverting the age intervals to identify the point at which the megafaunal populations began to decrease before full extinction, we used linear regression with breakpoints, estimated through lowess smoothing. The sources of 171 radiocarbon dates are, n = 6 this study; n = 24 from Araújo *et al*.^139^, n = 3 from Pino *et al*.^27^, n = 47 from Prado *et al*.^140^, n = 27 from Surovell *et al*.^122^, and n = 64 from Villavicencio *et al*.^119^. Duplicates were eliminated. The dates were ranked in quality from 11 to 17 by the original authors based on multiple factors (e.g., the kind of material dated, the association of the dated material with the taxon or event that was being dated, and the method of dating (AMS or standard)). Dates lower than the rank of 11 were excluded from this study because, as suggested by Barnosky and Lindsey^118^, lower-ranked dates are considered to be insufficiently robust. Megafaunal remains were excavated and analyzed under the supervision of M.P. at the Pilauco Lab and at the Universidad Austral de Chile in Valdivia.

#### Summed probability analyses

We followed the Bayesian technique from Anderson *et al*.^106^ that involves calibrating megafaunal radiocarbon dates and stacking the probability ranges using the SUM algorithm in OxCal v. 4.3.2. Major peaks and troughs are inferred to represent changing megafaunal population densities, i.e., greater numbers of dates are inferred to reflect larger populations, with fewer dates indicating smaller populations^106^. The advantage of this technique is that it relies on the entire range of uncertainties for the calibrated dates.

## Supplementary information

Supplementary Information

## Data Availability

all relevant data are available in this contribution. Sedimentary sample aliquots are available from Mario Pino, email: mariopino@uach.cl.
