# Supplementary material for: Sedimentary record from Patagonia, southern Chile supports cosmic-impact triggering of biomass burning, climate change, and megafaunal extinctions at 12.8 ka
Source: Sci Rep. 2019 Mar 13;9:4413. doi: 10.1038/s41598-018-38089-y (PMC6416299; doi:10.1038/s41598-018-38089-y)
Supplement: Supplementary file 1 — Supplementary Information [file 41598_2018_38089_MOESM1_ESM.docx]

**Sedimentary record from Patagonia, southern Chile supports cosmic-impact triggering of biomass burning, climate change, and megafaunal extinctions at 12.8 ka**

Mario Pino, Ana M. Abarzúa, Giselle Astorga, Alejandra Martel-Cea, Nathalie Cossio-Montecinos, R. Ximena Navarro, Maria Paz Lira, Rafael Labarca, Malcolm A. LeCompte, Victor Adedeji, Christopher R. Moore, Ted E. Bunch, Charles Mooney, Wendy S. Wolbach, Allen West, James P. Kennett

**SUPPLEMENTARY INFORMATION:**

**Text S1. Previously reported evidence of YDB magnetic spherules.**

This study of the Pilauco sedimentary sequence identifies 6 groups of spherulitic particles:

Group 1: Melted YDB impact spherules with dendritic textures and/or vesicles;

Group 2: Melted Cr-rich YDB spherules;

Group 3: Melted glassy volcanic spherules with low-Fe content;

Group 4: Unmelted, authigenic framboids with numerous cube-like crystals, often with high sulfur content;

Group 5: Melted anthropogenic spherules in surface sediments;

Group 6: Unmelted, rounded, detrital grains often with high titanium content and visible facets.

For distinguishing characteristics of each type, see **Table S10**. These spherulitic objects cannot be distinguished from one another using a reflected-light microscope. Instead, they require the use of SEM-EDS and/or a microprobe, as specified by Firestone et al.^1^, who wrote on page 17-18 of their Supporting Information: “*spherules were either left whole or sectioned and given a microprobe polish for analysis by laser ablation or x-ray fluorescence (SEM/XRF).*”

Firestone et al.^1^ identified YDB peaks in impact-related spherules at 14 sites. Of 13 subsequent independent studies, 8 performed SEM-EDS and/or microprobe analyses and found a significant peak in the YDB, as predicted. The other 5 studies reported that “YDB spherules” are heterogeneously common throughout the sediment, and, therefore, cannot be impact-related. However, those studies either did not perform SEM-EDS analyses or performed them incorrectly, and therefore could not differentiate between anthropogenic, authigenic, volcanic, and impact-related spherules. Instead, they assumed that all spherulitic particles they found were high-temperature, melted “YDB spherules.”

Independent studies of YDB magnetic spherules:

**Performed SEM-EDS**. Baker et al.^2^ reported finding a YD-age site in Montana, writing that “*the black mat contains … unrusted iron micro-meteorites [native iron magnetic spherules]. SEM photos of iron micro-meteorites reveal fusion crusts, flow lines, and micro-impact craters--direct evidence for an extraterrestrial origin*.”

**Performed SEM-EDS, microprobe**. At Murray Springs, Arizona, Fayek et al.^3^ found that “*impact material contains iron oxide spherules (framboids) in a glassy iron-silica matrix, which is one indicator of a possible meteorite impact. ... Such a high formation temperature is only consistent with impact ... conditions.*” Because the framboids were encased in meltglass, they are inferred to have resulted from a cosmic impact.

**Performed SEM-EDS**. Ge et al.^4^ reported YD-age “*microtektite-like glassy spherules*” from 3 widely separated sites, in France, in the Caspian Sea, and in the Peruvian coastal desert. They state that the evidence “*supports an impact origin from an ejecta plume*.”

**No SEM-EDS**. Surovell et al.^5^ were unable to reproduce the spherule data of Firestone et al.^1^, and, instead, reported finding YDB spherules heterogeneously distributed throughout the sediment profiles at seven sites. They concluded that YDB spherules are common and not restricted to the YDB layer, and, therefore, cannot be impact-related. Their Methods section states that the group used an updated spherule protocol from Firestone et al.^1^ sent by one of the co-authors (A.W.) of this contribution. However, Surovell et al. did not conduct any SEM-EDS analyses of the candidates, as specified in the Firestone et al.^1^ protocol. Thus, it is unclear what this group found, but they likely misidentified some detrital grains and/or framboids as “YDB spherules.”

**Performed SEM-EDS**. In 2010, Laub^6^ investigated sediment samples from the Hiscock archaeological-paleontological site in western New York state. He reported that “*iron-rich spherules, 50-65 µm in diameter, were found in the Pleistocene horizon*” spanning the YD onset, as reported by Firestone et al.

**Performed SEM-EDS**. Mahaney et al.^7,8^ found in Venezuela “*a mixed assemblage … of Fe spherules*” in a YD-age layer “*with a frequency higher than chance occurrence.*” Mahaney et al.^8^ concluded that the “*new evidence … point tentatively to either an asteroid or comet event that reached far into South America*.”

**Microprobe; no SEM-EDS; flawed analyses**. Haynes et al.^9^ observed high concentrations of magnetic spherules in the YDB layer at Murray Springs, Arizona, consistent with the observations of Firestone et al.^1^ and Fayek et al.^3^ They also reported abundant spherules from modern alluvium and rooftops, concluding that their presence refutes the YDB hypothesis. They conducted SEM-EDS/microprobe analyses, but did not differentiate between YDB spherules, anthropogenic spherules, framboids, and detrital grains, and instead, assume that all were “YDB spherules.”. Many of these types are nearly ubiquitous in surface sediments.

**SEM-EDS; flawed analyses**. Pinter et al.^10^ reported on two sites in California at which “*magnetic spherules are heterogeneously distributed in sediments*”, and concluded that this refuted the YDB impact hypothesis. Although this group acquired SEM images to support their argument, they presented only images of unmelted framboids and unmelted detrital grains and showed no SEM images of melted, dendritic YDB spherules, thus calling into question their conclusions that they observed abundant “YDB spherules” at all.

**Performed SEM-EDS, microprobe**. Wu et al.^11^ reported YDB spherules from two sites in North America and found evidence of high-temperature melting under low-oxygen conditions, as at Pilauco. They concluded that “*the [Fe-rich] spherules could be generated in a meteorite impact.*”

**No SEM-EDS**. Pigati et al.^12^ confirmed a YDB peak in spherules at Murray Springs, Arizona, as previously reported by Firestone et al.^1^ and Fayek et al.^3^ They also reported multiple non-YDB spherule peaks at Chilean sites and claimed that this refuted the YDB impact hypothesis. The Chilean sites, however, are known to contain abundant volcanic spherules^13^. Because Pigati et al. did not conduct any SEM-EDS analyses, they assumed that the abundant spherules they found near volcanoes were "YDB spherules", thus calling their conclusions into question.

**Performed SEM-EDS**. LeCompte et al.^14^ compared the results of Firestone et al.^1^ and Surovell et al.^5^ at two sites: Blackwater Draw, NM and Topper, SC. They reported that their “*spherule abundances are consistent with those of Firestone et al.^1^ and inconsistent with the results of Surovell et al.*” They concluded that Surovell et al. were unable to reproduce the results of Firestone et al. “*primarily due to their failure to adhere to the [Firestone] protocol*”, mainly by omitting SEM-EDS analyses.

**Mixed SEM-EDS**. In a blind test, Holliday et al.^15^ reported that both participants found “YDB spherules” in non-YDB sections of a sedimentary profile in Texas, but one participant performed no SEM-EDS analyses to confirm that claim. The other participant used SEM EDS to identify found an unpublished peak in spherules in the YDB layer.

**Performed SEM-EDS**. Andronikov et al.^16^ reported peak abundances of spherules in the YDB layer at a site at Blackwater Draw, New Mexico. SEM-EDS analyses showed that some spherules were highly enriched in iridium and platinum. Andronikov et al.^16^ stated that “*Our observations confirm those made by Firestone et al.^1^ and LeCompte et al.^14^*,” but they contradicted the previously reported lack of a spherule peak by Surovell et al.^5^

In summary, the majority of independent studies (8 of 13) that conducted SEM-EDS analyses confirmed the presence of YDB spherules in layers that date to ~12,800 cal BP. The other five studies did not conduct SEM-EDS correctly and could not confirm the presence of YDB spherules.

**Text S2. Background: Paleoclimate and Biota.**

Pilauco is located in southern Chile between ~38° to 56° S and the region is largely influenced by westerly winds of the Southern Hemisphere. Presently, the region spans three major vegetation zones: (1) from 37º 45’ S to 43º 20’ S, including Pilauco, where the vegetation corresponds to the Valdivian rainforest that is replaced farther south by; (2) the North Patagonian forest (to 47º 30’ S), and then, in turn, by (3) the sub-Antarctic Flora in association with the Magellanic Moorland (to 55º30’ S)^17-19^. This meridional zonation in vegetation is largely due to a strong precipitation gradient with annual values ranging from 1500 mm at 38º S to 4500 mm at 47° S. In contrast, the annual atmospheric temperature range over these same latitudes is only 2 °C^20^.

During the late Quaternary, the meridional range of these three vegetation provinces was likely affected by multi-millennial changes in the latitudinal position and/or the intensity of the westerly winds, causing significant variations in precipitation and dryness. Additionally, it has been postulated that during the Last Glacial Maximum (LGM; from ~23,000 to 17,500 cal BP^21,22^) the westerly winds may have intensified and expanded towards the equator thus bringing lower temperatures and higher precipitation rates to the Pilauco area^22-25^. Also, there is evidence of altitudinal descent and/or meridional advances in vegetation that occurred after the LGM^23^. This evidence includes presence of the Magellanic Moorland circa Llanquihue (41°S) and Chiloé (42°S) between 17,500 to 16,000 cal BP^22,26-28^, and presence of the evergreen tree, *Araucaria araucana* in lowland areas of the Araucanía Region (38º S) between 26,000 and 16,000 cal BP^29^.

Several pollen records from northern Patagonia exhibit a vegetation shift, which began at ~17,500 cal BP. This shift was from cool rainforests dominated by cold-resistant *Nothofagus dombeyi*-type and grasslands (Poaceae and Ericaceae) to a warmer thermophilous vegetation. Also, expansions of the Myrtaceae family and the arboreal vine *Hydrangea serratifolia*, at ~16,000 and 14,000 cal BP respectively, reflect this warming trend^30-32^. In the Chiloé area to the south, most early deglacial pollen records are dominated by Magellanic Moorland species that typically reflect cold, wet conditions. Pollen records from Lago Lepué in Chiloé^32^ and from the Chilean Lake District^31,33-35^ also exhibit increased Podocarpaceae taxa (southern conifers) indicating cooling with increased precipitation until ~13,000 cal BP. Between ~13,000 to 11,600 cal BP most regional pollen records indicate an increased magnitude and seasonality in precipitation. This interval is also marked by increased charcoal concentrations reflecting a significant increase in biomass burning^32,36^.

In northwestern Patagonia (38º to 42º S) during the last glacial termination, glaciers occupied both sides of the Andes Cordillera and part of the Intermediate Depression, replacing extensive areas of vegetation^37^. At ~17,500 cal yr BP, the glaciers experienced a rapid recession in lowland areas, and within a thousand years, temperatures increased reaching near-modern values, which promoted the recolonization by vegetation of areas disrupted by glacial activity^30,38^. Beginning at ~14,400 cal BP, multiple regional climate records in lake sediments (~40° S) show that the warm climate became colder during the Antarctic Cold Reversal^39-42^, locally known as the Huelmo-Mascardi Cold Reversal^31,43-45^.

**Text S3. Sedimentology**

The earliest sedimentological interpretation of the Pilauco section was that its sandy sediments and gravel were associated with fluvial plain deposits within a former oxbow lake^46^. It was inferred that the gravel was transported as rolling bed load to the south of the old Damas River’s main channel and into a swamp. Later, Pino et al.^46^ noted that surfaces of the aphanitic basalt boulders and pebbles had no impact marks, contradicting the previous interpretation that there had been high-velocity transport. In the grids excavated after 2013, new stratigraphic observations indicate that in units PB-7 through PB-9 there are no sedimentary structures suggesting fluvial deposition (e.g., imbrication and cross-bedded laminations). Pino et al.^47^ performed sand grain-size analysis (mean grain size, sorting, skewness, and kurtosis) and discriminant analyses on 27 samples from units PB-7 and PB-1, the latter (San Pablo Unit) having been collected from the hill bordering the site to the north. These results indicate that the siliciclastic sediments of both units are indistinguishable. This means that the sediments, dominated by very coarse and coarse sand, were not high-energy river deposits, but instead resulted from colluvial processes (gravitational and/or water transport from the northern hills). The laminar dip in unit PB-9 indicates that the sediment was transported downslope from the north. The textures suggest that, at times, stratigraphic deposition of this unit was primarily driven by gravity (boulders of volcanic tuff in a fine matrix, see **Figure S4**), and at other times by water transport/deposition (probably intense rain) that formed ponds in which laminated sediments accumulated (see **Figure S4**).

River deposits typically exhibit a close relationship between concentrations of gravel and very coarse sand. Linear regression analysis on these parameters revealed no statistically significant relationship between the gravel and very coarse sand fractions. This indicates that the abundance of gravel is not related to colluviation from the nearby San Pablo Unit, and also reinforces the idea that river processes did not deposit unit PB-7.

Colluviation explains the coexistence of a sand matrix, carbon organic matter, and gravel clasts “floating” in a mixture of sand and mud. After deposition of the YDB layer in unit PB-8, the swamp was probably seasonally dry, especially during summers, as indicated by the presence of carabids and dung beetles^48^. In unit PB-9, large amounts of volcanoclastic cobbles and pebbles were deposited during intense erosional processes on the northern hills following forest fires.

The basal unit studied in the Pilauco stratigraphic sequence is PB-6, an unconsolidated sediment containing well-rounded pebbles to boulders of plutonic and volcanic Andean rocks, ranging from 1 to 15 cm in diameter. Unit PB-6 contains ~10% organic matter with ~60% sand (sieved from 2000 to 62.5 μm) and ~30% mud (<62.5 μm) (**Table S2, S3**).

The next higher unit, PB-7, is an organic-rich sand composed of isolated, poorly sorted colluvial volcanoclastics, ranging in size up to 7 cm (percentage not quantified) with the remainder composed of 70.7% sandy matrix (2000 to 62.5 μm), 16.3% mud (<62.5 μm), and 12.9% fine organic material, as determined by loss-on-ignition values. The sediment’s color is brownish-black (Munsell scale: 10YR 3/1) (**Table S2, S3**). Unit PB-7 is separated from unit PB-6 by an abrupt and variably inclined boundary, and contains most of the extinct megafauna remains (e.g., fossil remains of the Gomphotheriidae), bones of extant micromammals, numerous vegetal and invertebrate remains, and lithic artifacts.

Unit PB-8 is composed of organic-rich mudstones separated by thin, muddy laminae, similar to unit PB-7. The unit is slightly brown in color (2.5Y 4/2) (**Table S2, S3**). Both units were deposited in a seasonal swamp with colluvial contributions of volcanoclastic sand and gravel. The upper boundary of unit PB-8 dips about 15º to the south near the northern hill (grids 6AD to 7AD) and is nearly horizontal between grids 7AD and 10AD. This unit contains fewer mammal fossils and lithic artifacts than unit PB-7 below it, and although fossil remains of Gomphotheriidae are not recorded in this unit, it contains the youngest horse coprolite found at the site^46,47^.

At Pilauco, the key stratigraphic feature is the PB-8/PB-9 boundary, marking major changes in the environment and in sedimentation. Immediately above that boundary there is a large accumulation of wood fragments, frequently found along a sharply defined, undulating contact (**Figure** **S2, S3, S4**). This boundary marks the sudden onset of higher sedimentation rates and increased transport of sediment as indicated by the presence of large blocks from the northern hills associated with fine sediment laminae, thin lenses, and lag deposits of material sorted by aeolian, fluvial, and other natural processes (**Figure** **S4**).

Unit PB-9 is largely a relatively fine-grained, black, organic-rich peat (mean matrix color 2.5Y 2/0) (**Figure 2**) that is lithologically and environmentally much more variable than units PB-7 and PB-8. This unit contains numerous southward-dipping lenses with varying concentrations of organic and inorganic material. The peat includes abundant yellow volcanic tufa fragments derived from volcanoclastic layers in the northern hills, as well as decimetric wood and charcoal fragments. Unit PB-9 is composed of a mixture of 74.4% sandy matrix, 16.2% fine mud, and 9.4% fine-grained organic material. The percentage of the fine sediment fraction (<106 µm) doubled above the PB-8/PB-9 boundary from ~30% to ~60% before gradually increasing to ~70% (**Figure S5; Table S2, S3**).

The principal constituent of units PB-6 through PB-9 is sand (>60%) that acts as a matrix (**Table S2, S3**). Textural analysis of sediment samples from the top of unit PB-6 to the top of PB-9 (grid 14AD, 255 samples, 326 to 581 cm altitude) indicates that gravel (mostly volcanoclastic) constitutes between 12 to 22% of the total inorganic sedimentary fraction. Because the samples were only 5 cm wide, the concentrations of gravel are random rather than representative, and so they were not considered in the textural description. The units were subdivided into subunits A through F (**Figure 2**), where A, B and C have sand concentrations of 63%, 58%, and 60%, respectively, and mud concentrations of 37%, 42%, and 40%, respectively (gravel not quantified). These subunits differ mostly in their concentrations of microscopic charcoal. The most conspicuous subunit (D) is siliciclastic, containing the highest concentration of tuff fragments deposited by colluvial processes from the northern hills. The subunit’s composition is 15% blocks, 66% gravel, 11% sand, and 8% mud. Lenses E and F are dominated by fine local volcanoclastic material at 88% and 95%, respectively, with the remainder as organic material. Granulometric analyses indicate lens E is composed of 10% sand and 90% mud; lens F is 4% sand and 96% mud (**Table S2, S3**). In these lenses, the dark red and grey colors (10R 3/6 and 5Y 5/1) result from varying degrees of oxidation/reduction of organic matter. A young organic soil caps the sequence.

The percentage of organic matter (~10%) in units PB-7 through PB-9 was calculated by loss-on-ignition. However, this value does not really represent its abundance because the low carbon concentrations of ~0.7 g cm^-3^ (relative to mineral concentrations of 2.6 g cm^-3^) would be ~3 times greater if volume is considered. Organic matter is estimated to average ~30% by volume, which explains the plastic appearance of these units (**Table S2, S3**).

**Text S4. Background on human lithics and artifacts.**

At most South American sites older than 11,700 cal BP, the most commonly recorded cultural remains are non-formal lithic materials. Arguments about the earliest age of occupation generally have focused on whether the ages of the sites are correct and whether the cultural remains represent the development of local lithic technologies (cf. references in Bryan and Gruhn^49^ and Gnecco and Aceituno^50^). Most of the discussion is centered on the comparison of area lithics with bifacial technology, dating from the Pleistocene-Holocene boundary^51,52^. However, recent studies provide new evidence of Pleistocene sites dominated by unifacial lithics^53,54^. At Pleistocene settlements across South America, both unifacial and bifacial lithic materials were derived mainly from locally sourced materials^55^. This trend shows a strong shift at the beginning of the Holocene, as in North America, where different styles of points and other bifacial tools, produced from more diverse raw materials, became more frequent in the archaeological record^52^. We infer that the transition from Clovis to Folsom points near the YD onset in North America is similar to the transition that occurred at the same time in South America from the Edge-Trimmed Unifacial Tradition (with few examples of bifacial artifacts) to the well-developed Bifacial Tradition^54^.

**Text S5. Earlier South American impact events.**

In South America, at least two large cosmic impact events are associated with major faunal extinctions, similar to those at ~12.8 ka. The first, the 66-Ma-old K-T impact in Yucatán, triggered the worldwide extinction of ~75% of all extant species^56^ and caused abrupt, severe climate change.

During the middle Pliocene, around 3.3 million years ago, a major cosmic event deposited impact-related meltglass across a large part of eastern Argentina^57^. This impact event is associated with significant extinctions of terrestrial fauna in the Pampas region of Argentina, involving ~37% of all genera and ~53% of all species^58^. This event was also coeval with an abrupt change in the deep-sea isotopic record that reflects an abrupt disruption of climate and ocean circulation^57^.

Thus, cosmic impacts are known to have previously triggered extinctions and abrupt climate change in South America, similar to those at the YD onset.

**SUPPLEMENTARY INFORMATION: Figures**


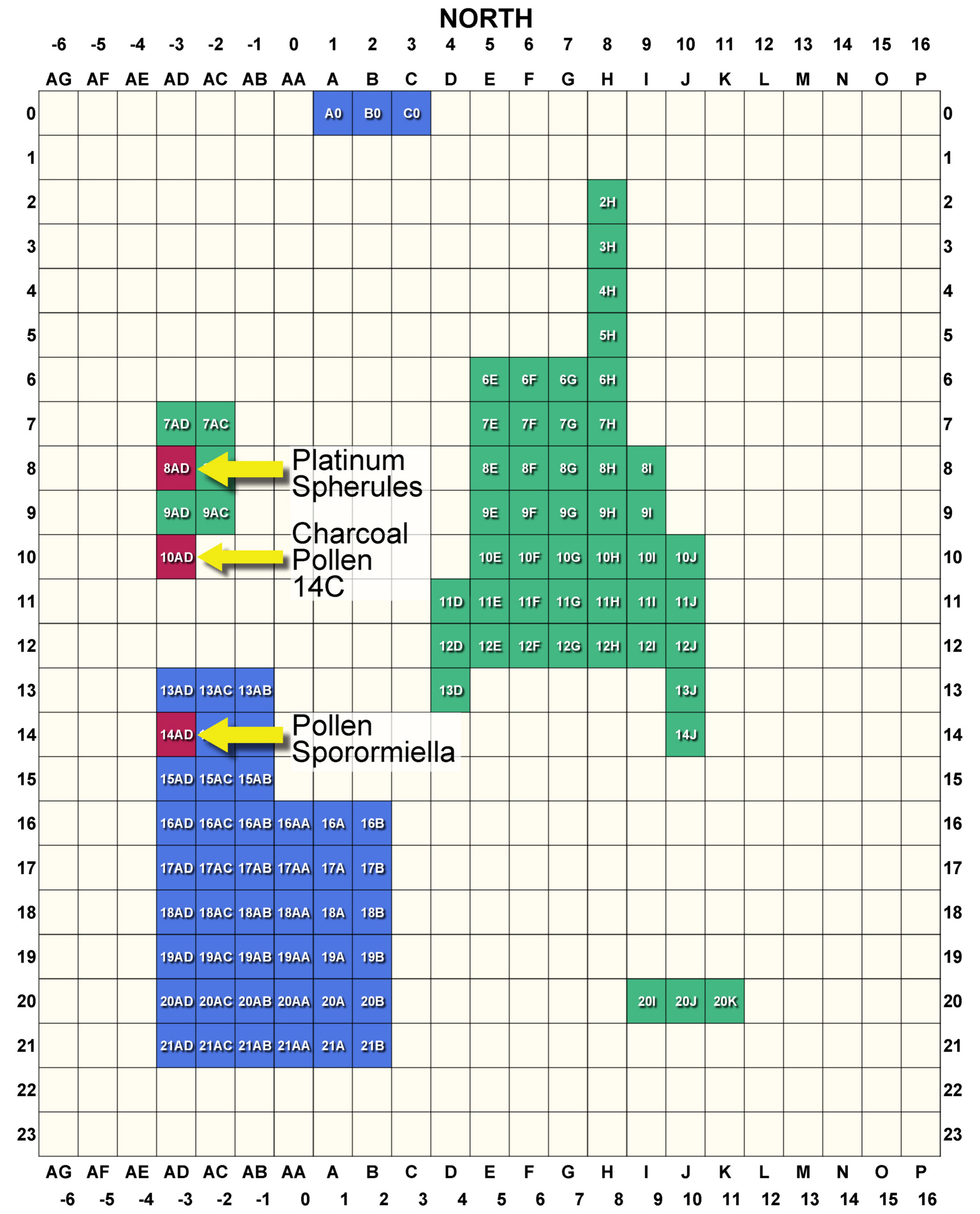


**Figure S1. Excavation grids at Pilauco.** Blocks in red represent those areas of the excavation that contributed data to this paper. Grid 8AD was investigated for platinum and magnetic spherules; Grid 10AC was sampled for charcoal, pollen, and radiocarbon dating (in this study and previously^46^); and Grid 14AD was investigated for pollen and *Sporormiella* spores.


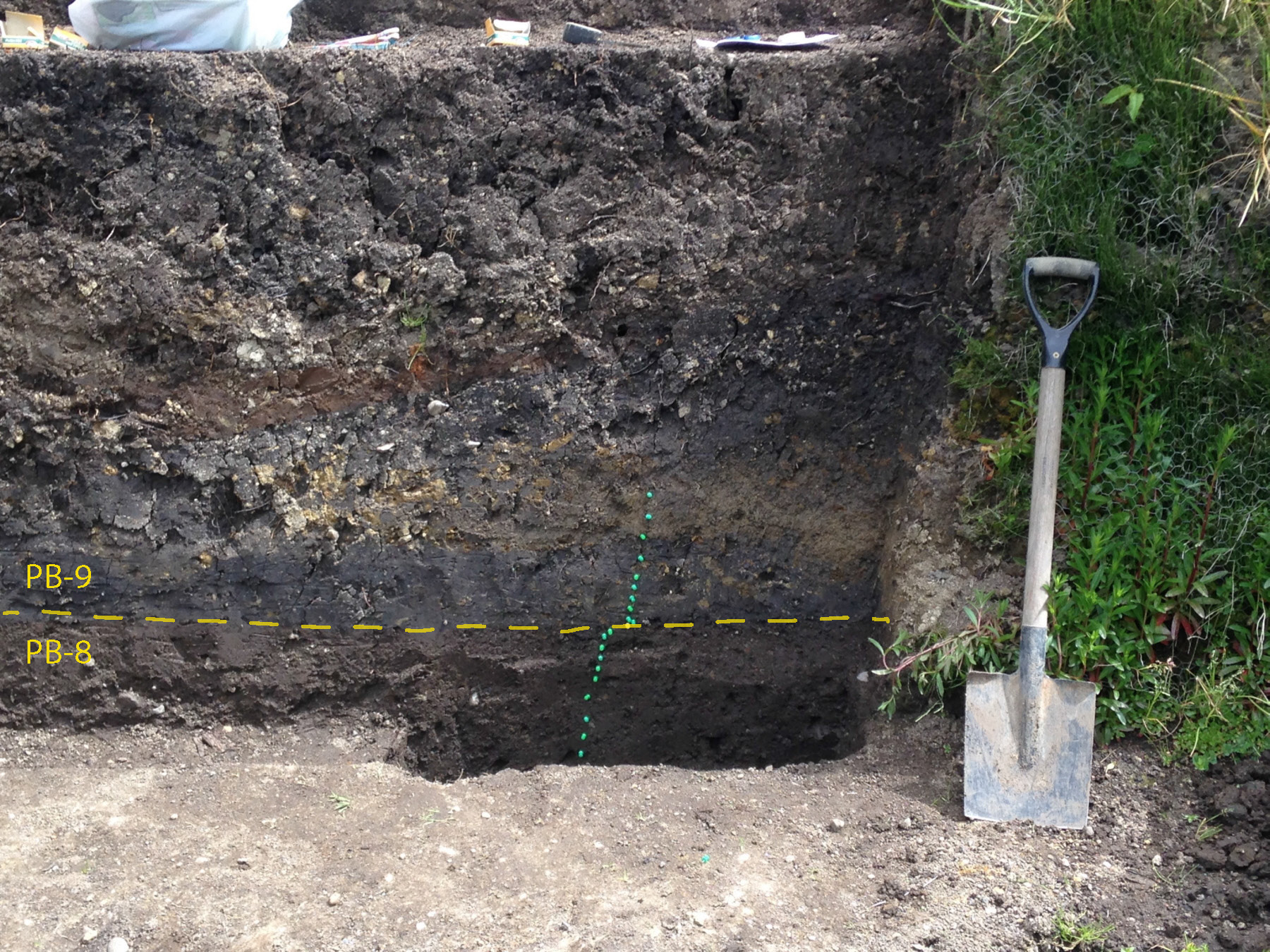


**Figure S2.** Sampling location in grid 8AD. Green pins indicate sampling locations.


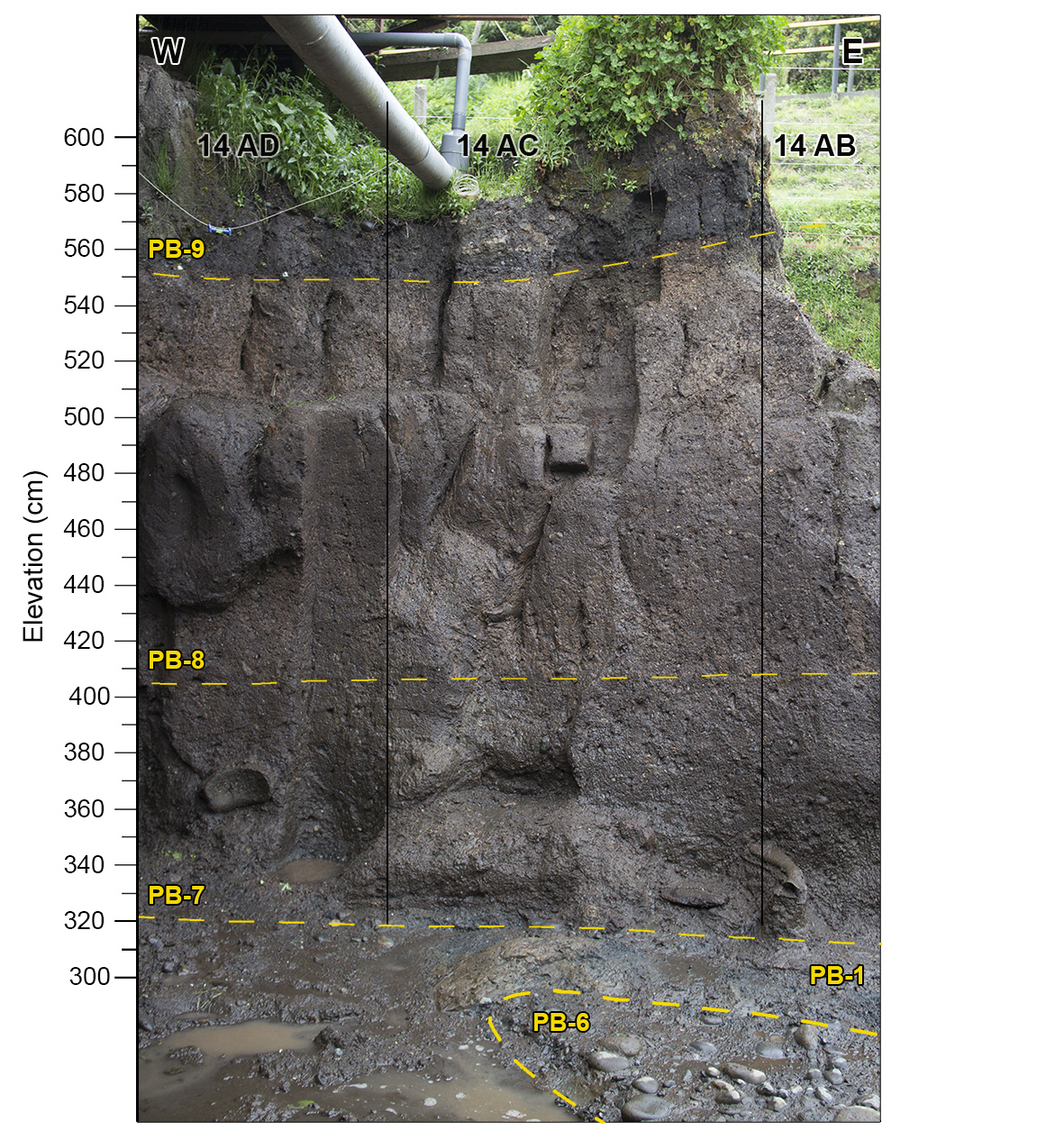


**Figure S3.** Image of sampling wall for grids 14AD through 14AB.


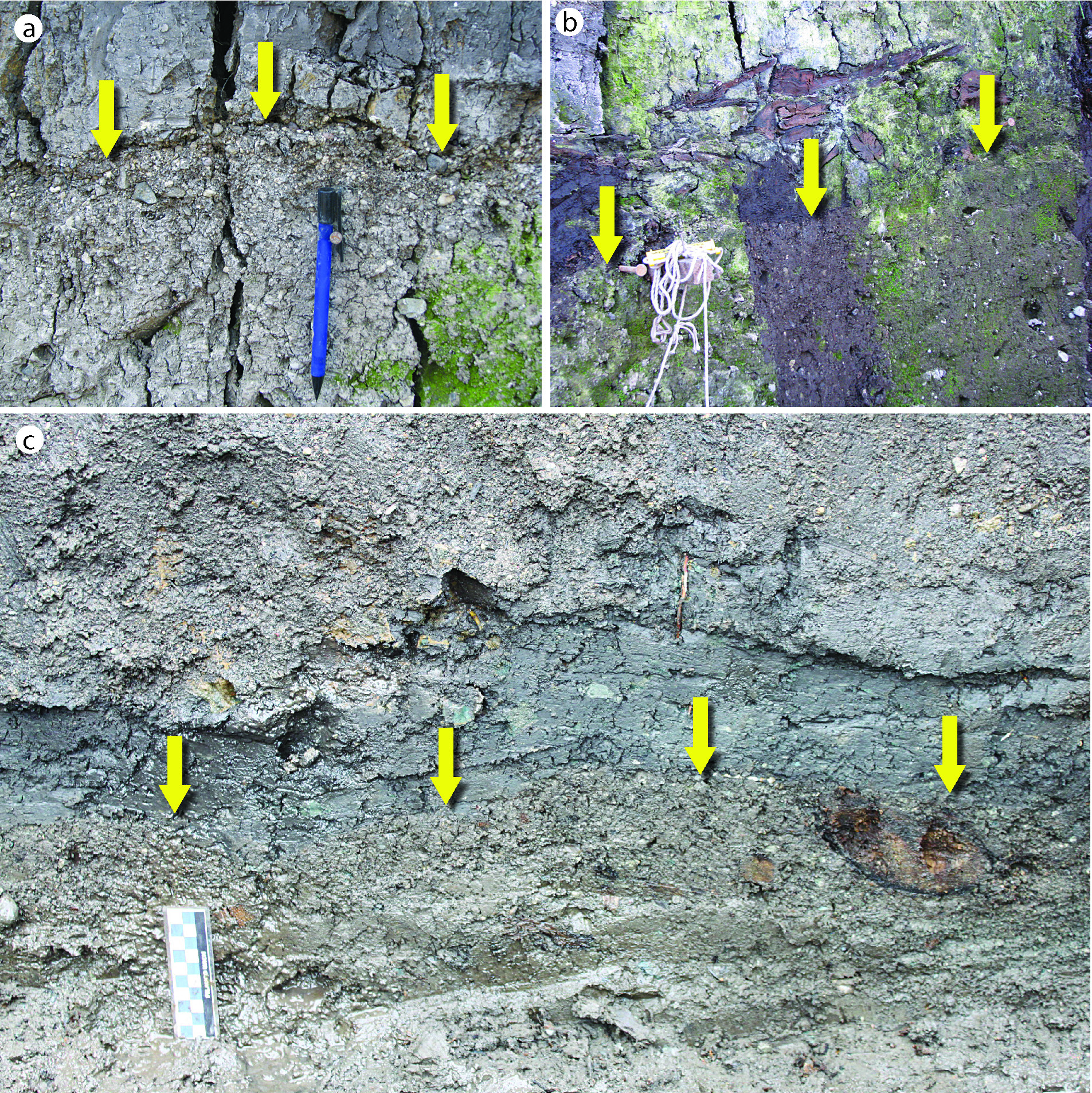


**Figure S4. Sediment boundary types.** Three types of PB-8/PB9 boundaries marked by yellow arrows. ***a)*** A gravel lag deposit; ***b)*** wood cluster; and ***c)*** sharp, undulating stratigraphic break.


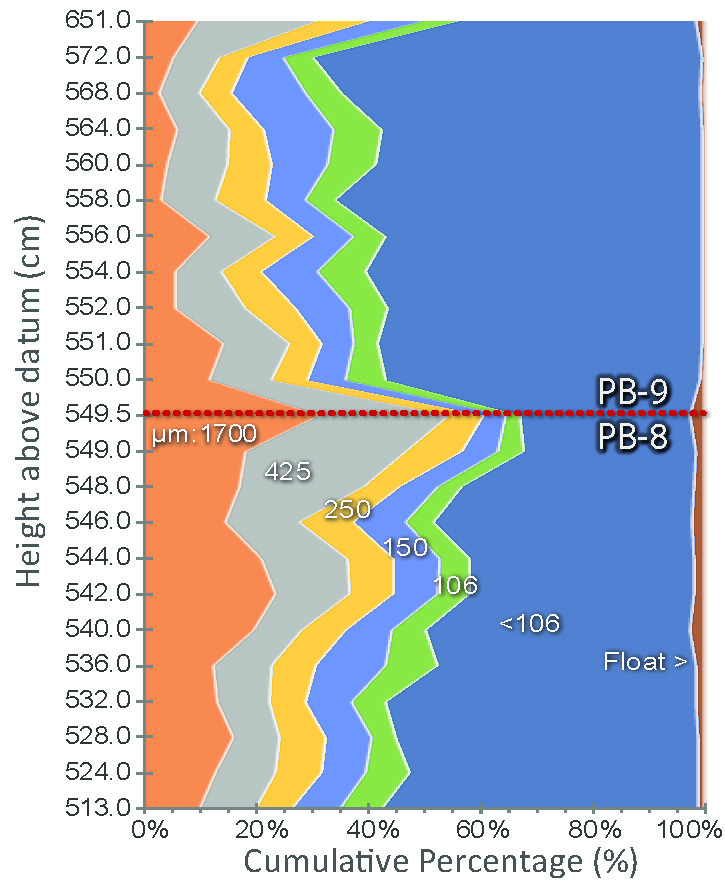


**Figure S5. Sediment grain size analysis.** The coarseness of sediment increases up to the YDB layer at the PB-8/PB-9 boundary. At that level, the size of the fine fraction <106 µm doubled to ~60% before gradually rising further to ~70%. This shift corresponds to climate change, an increase in biomass burning, and the resulting denuded landscape that, in turn, produced more fine-grained colluvium.


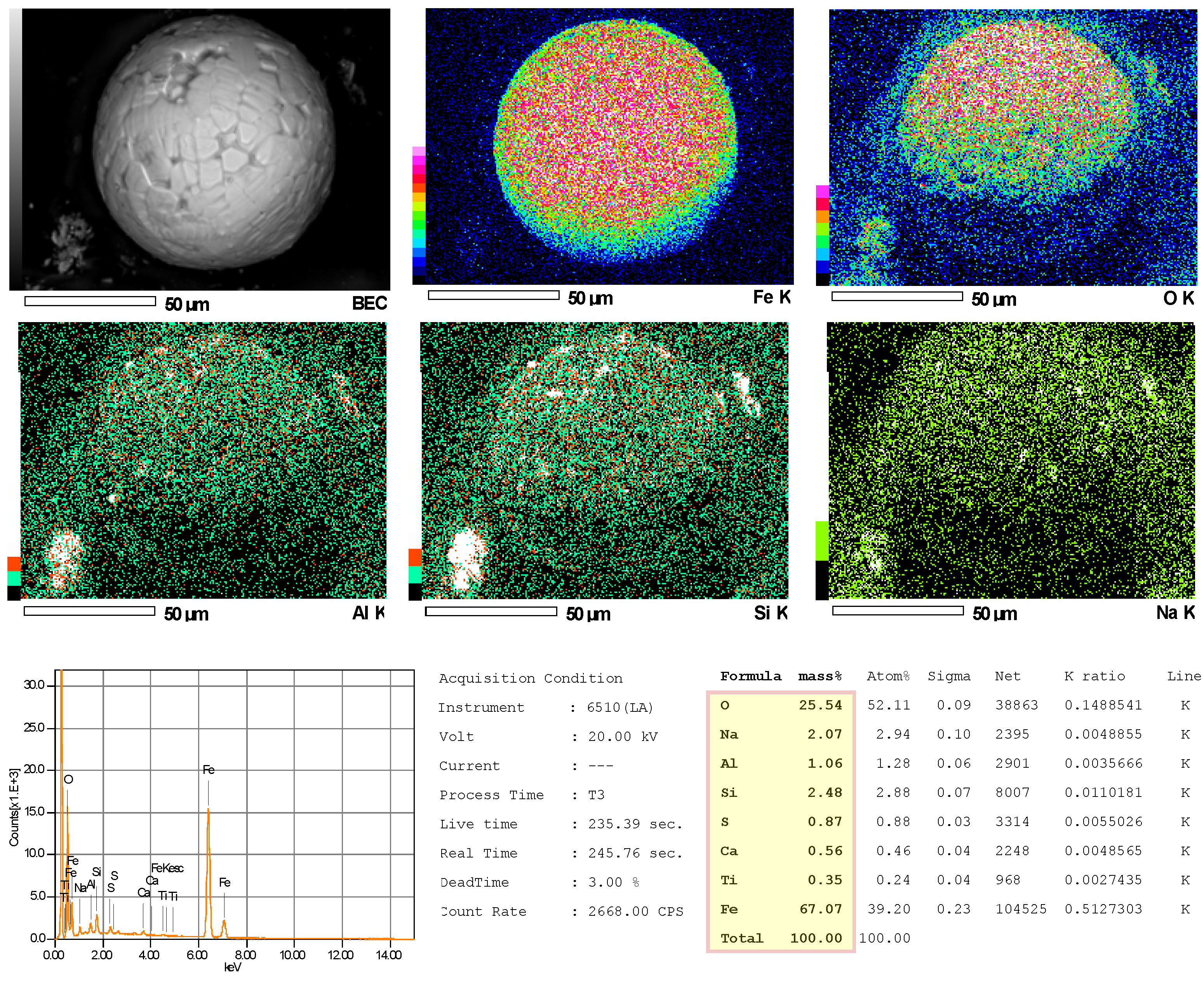


**Figure S6**. **SEM-EDS elemental map of YDB spherule Y6.** SEM image of YDB spherule shown in Figure 5b of main manuscript. SEM-EDS chemistry (**Table S7)** shows that spherule is composed of nearly all FeO. It contains only minor amounts of other elements, most of which are probable surface contamination, such as the white areas in lower row of images above.


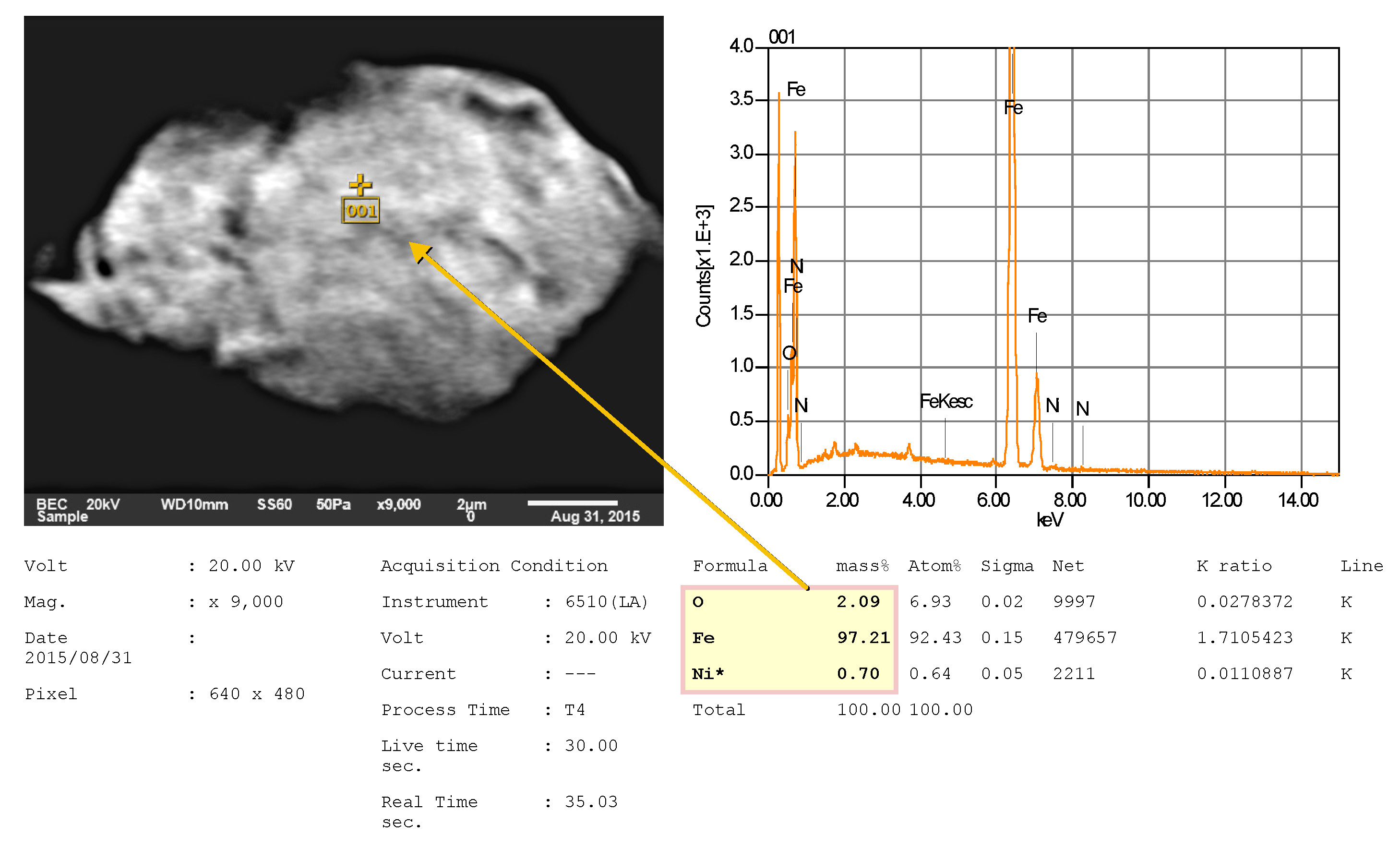


**Figure S7.** SEM-EDS of 11-μm-long, irregularly-shaped, native iron grain with >97% Fe and ~2% oxygen. Morphology indicates high-temperature melting under reducing conditions. The grain contains a small amount of Ni, possibly of extraterrestrial origin; alternately, the Ni content could have resulted from melting of Ni-bearing magnetite. Either way, this grain is not a product of natural terrestrial processes, but could have been melted at high-temperature during a cosmic impact event. From the 552-cm YDB layer.


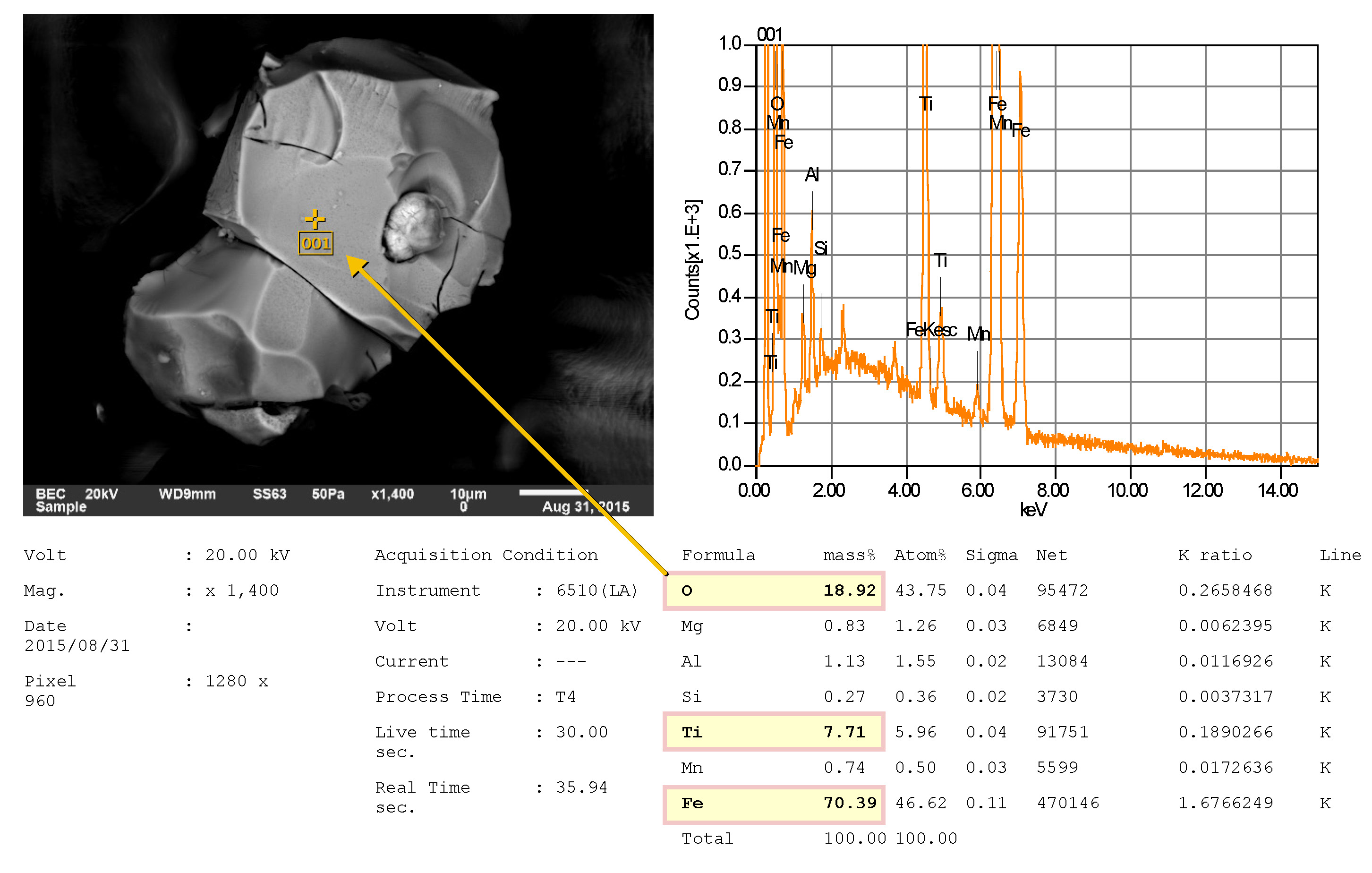


**Figure S8.** SEM-EDS of 65-μm-long titano-magnetite grain with a reduced oxygen content of ~19%, Fe content of ~70%, and Ti content of ~8%. From the 552-cm YDB layer.


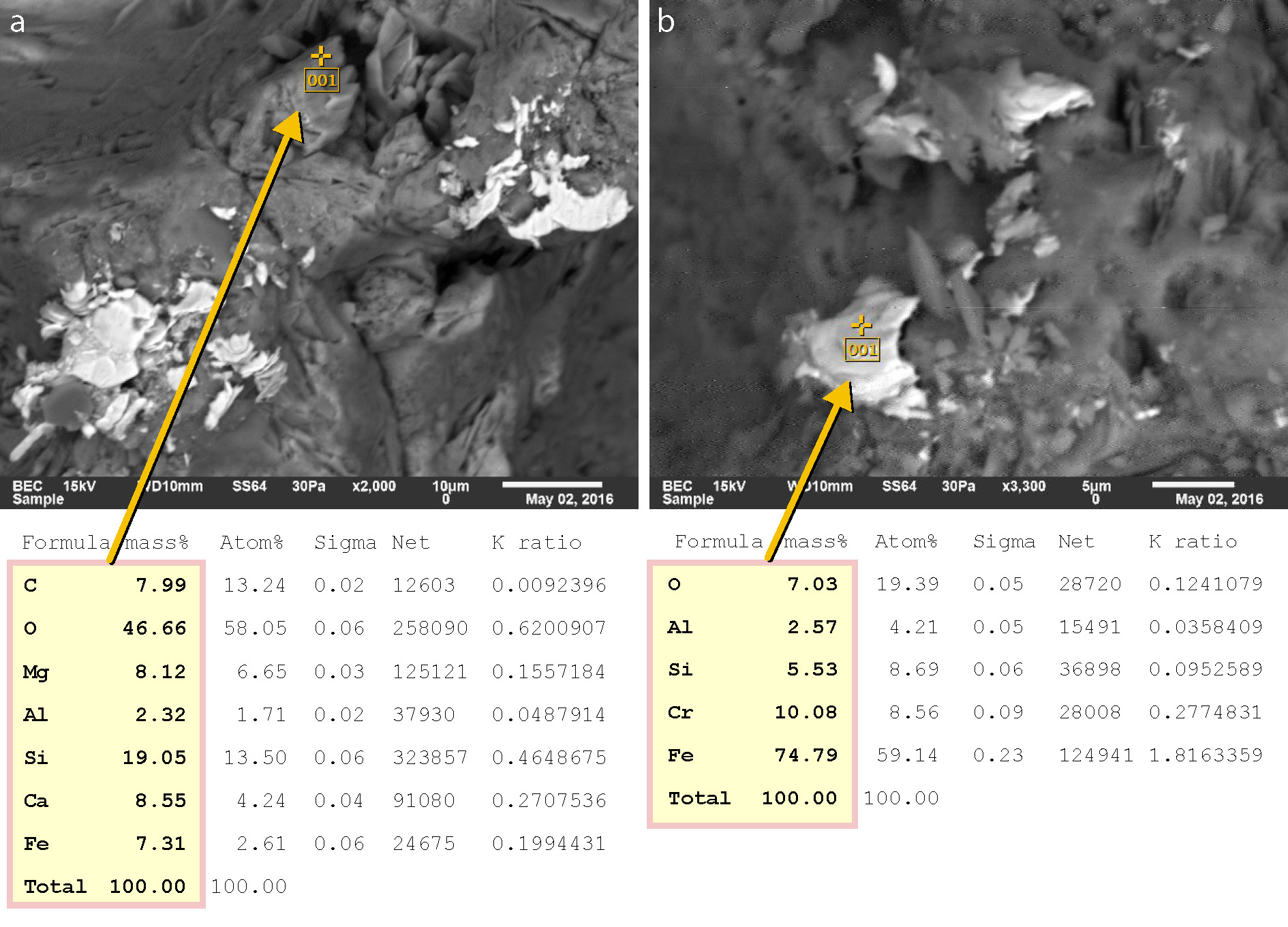


**Figure S9. Basaltic matrix and chromium-magnetite inclusions. *a)*** Spot SEM-EDS at yellow arrow indicates basaltic glass composed mainly of Si, Ca, Mg, Fe, and O. Ca is a typical component in Andean lavas whose origin is the subduction-arc complex along the Pacific coast of South America. The volcanic matrix contains no detectable chromium. ***b)*** Spot SEM-EDS at yellow arrow indicates bright metallic inclusions of chromium magnetite, composed of ~75% iron and ~10% chromium. There is no indication of rapid melting and quenching of these plate-like inclusions. Instead, they crystallized slowly from molten magma.


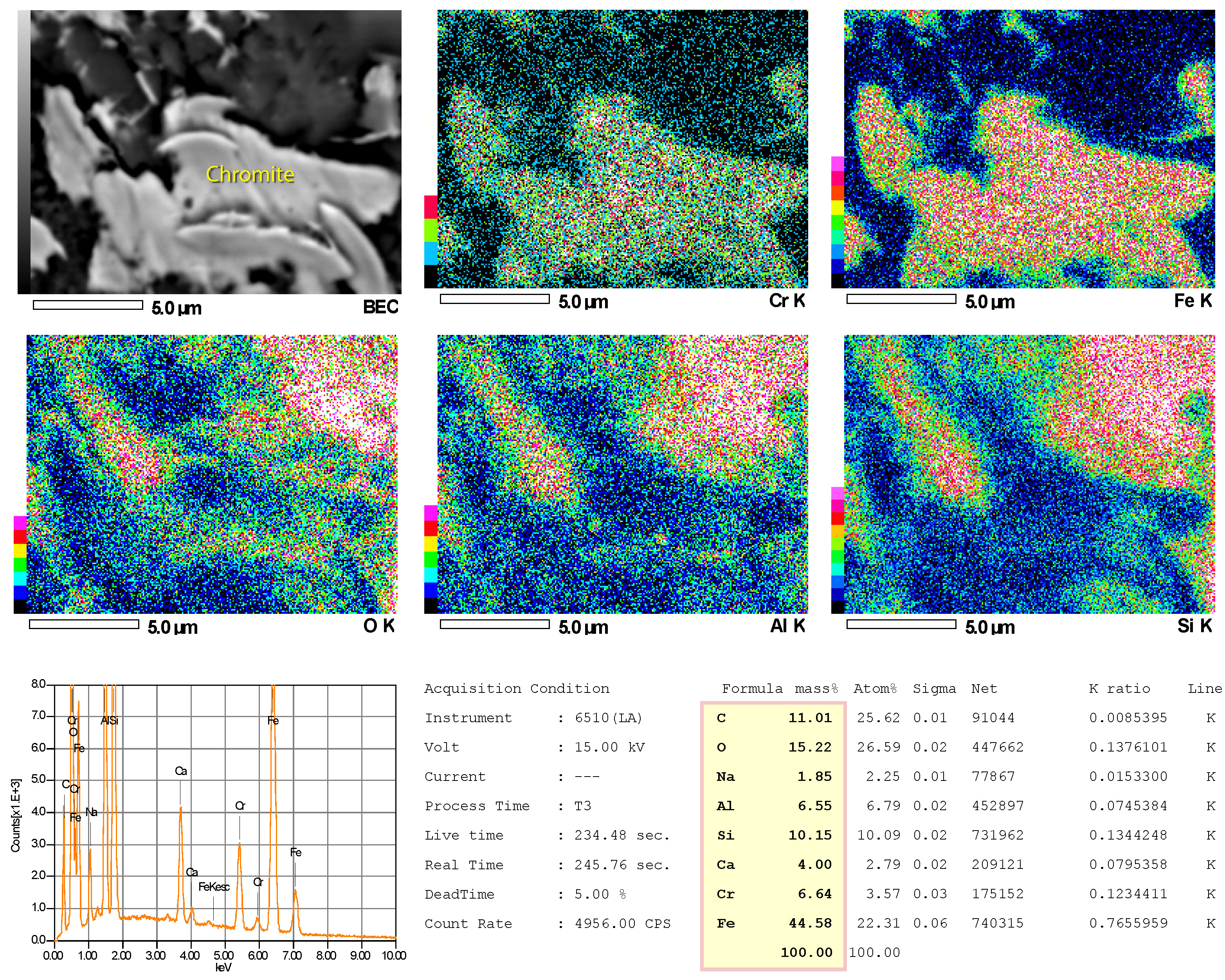


**Figure S10. SEM-EDS elemental map of chromium magnetite.** Analysis shows that bright areas in the upper left panel are metallic inclusions composed almost exclusively of chromium magnetite.


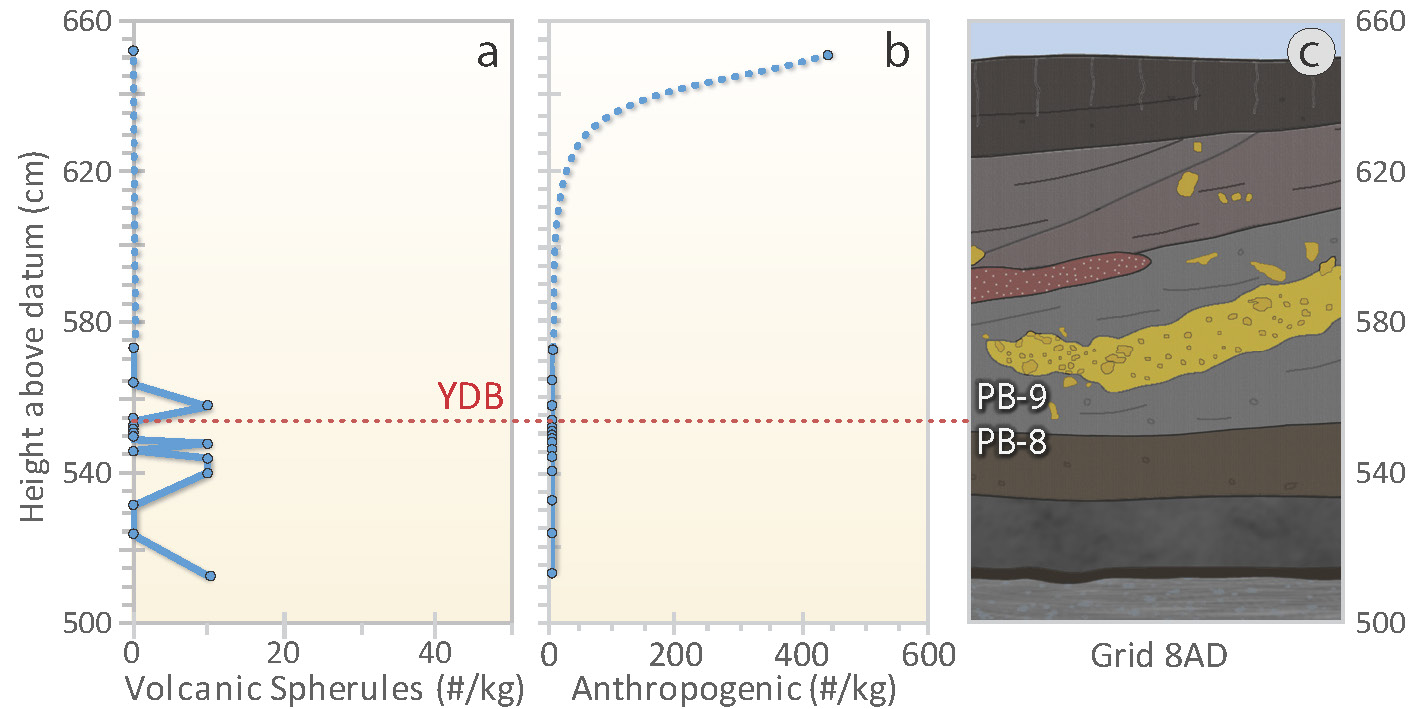


**Figure S11. Volcanic and anthropogenic spherules.** ***a)*** Volcanic spherules are randomly distributed throughout the profile in low quantities, ranging from 0 to ~10 spherules/kg. None were found in the YDB layer. ***b)*** Anthropogenic spherules are present only at the surface, and not in other samples. ***c)*** Stratigraphic profile of grid 8AD, where volcanic and anthropogenic spherules were collected.


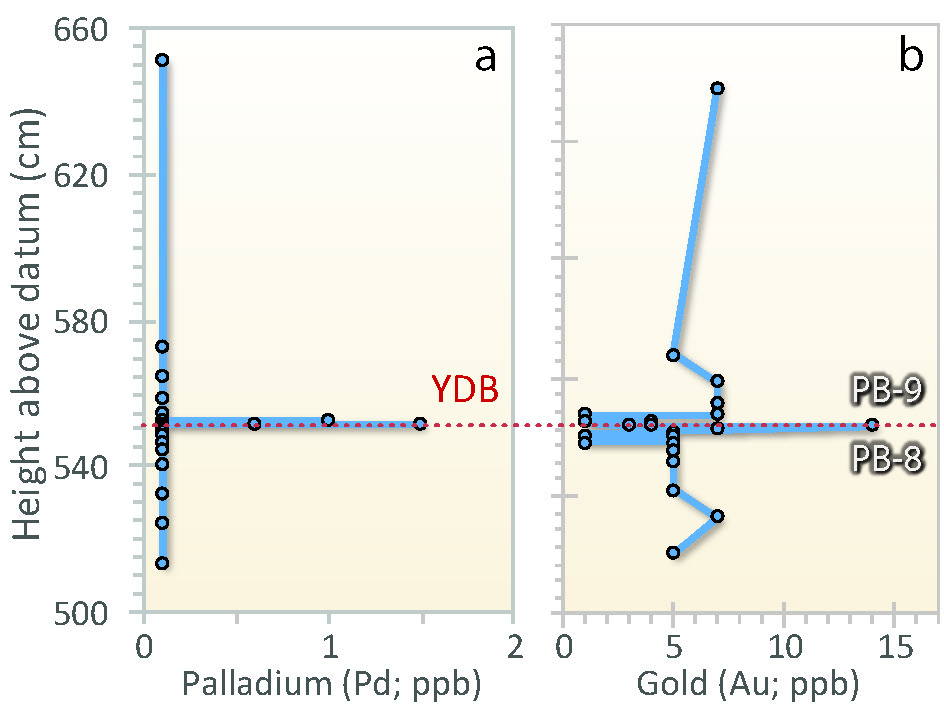


**Figure S12. Palladium and gold concentrations.** ***a)*** Palladium (Pd) concentrations are anomalously high at the YDB layer. ***b)*** Gold (Au) concentrations are also anomalously high at the YDB layer. The concentrations of Pt, Pd, and Au are much higher than background concentrations, suggesting a non-local source of these elements.

**SUPPLEMENTARY INFORMATION: Tables**

**Table S1. Age span of YDB sequences previously investigated.** The time-series samples analyzed from 24 YDB sites have a mean age span of 7,016 calendar years with a maximum span of ~21,375 calendar years. The age of sediment samples at Pilauco span 5,719 calendar years. Sampling in nearly all these studies was discontinuous, except across the YDB layer, leading some to argue that this makes it difficult to determine whether the inferred impact assemblage is unique to the entire record.

**Table S2. Sedimentology.** Textural parameters of units PB-7, PB-8 and PB-9 exposed in grid 14AD. Weight percentages of sand (sieved from 2000 to 62.5 μm), mud (<62.5 μm) and organic matter (whole sample, from LOI) for units PB-7, PB-8 and PB-9, grid AD14. The percentages were calculated with the exclusion of gravel (see main manuscript).

**Table S3. Granulometry for Lenses A-F.** See legend in **Figure 2** in main manuscript.

**Table S4. Radiocarbon dates from Pilauco.** Calibrated using Southern Hemisphere curve, SHCal13, within OxCal, version 4.3.2.

**Table S5. Bayesian age calculations.** Green line represents YDB at PB-8/PB-9 boundary at ~12,770 cal yr BP. Calculated using OxCal 4.3.3. with SHCal13 calibration curve.

**Table S6. Pilauco spherule groups.** Elevations and abundances of five groups of spherules. Group 6, detrital grains, is not shown.

**Table S7. SEM-EDS analyses of Pilauco spherules.** “Y” represents Group 1 YDB spherules; “C” are Group 2 Cr-rich spherules; “B” are Group 3 volcanic spherules; and “A” series represent Group 5 anthropogenic spherules. Composition of Group 4 framboids are discussed in the main manuscript.

**Table S8. Characteristics of spherulitic particles**. Categorized by geochemistry, morphology, texture, and level. Gold highlighted areas indicate some characteristics used to differentiate types.

**Table S9. Platinum (Pt), palladium (Pd), and gold (Au) concentrations by unit and elevation (cm).**

**Table S10.** **Distribution of seeds and cuticles**. Categorized by whether abundances were higher pre-YDB (blue), higher post-YDB (orange), or remained mostly the same throughout the profile. Red line represents PB-8/PRB-9 YDB boundary.

**Table S11.** Negative binomial generalised regression models calculated using the number of seeds present in units PB-8 and PB-9 at the Pilauco site. Model 1 refers to the complete model including an interaction term between the explanatory variables layer and sample size (model with the highest Akaike information criterion (AIC)); Model 2 is the additive model without the interaction term; Model 3 is the reduced model containing only the seed source (“Layer”) as the explanatory variable, and it was the best model based on lower AIC.

**Table S12. Distribution of pollen from grid 10AD.** Columns marked on blue are pre-YDB pollen samples (548-550 cm); columns marked in red are post-YDB (550-557 cm). Red line indicates PB-8/PB-9 boundary. Post/Pre-YDB Ratio is the relation between Post-YDB and Pre-YDB mean pollen/spores abundances (%). Ratios > 1 mean the increase of the taxa after YDB. Ratios <1 means the decrease of the taxa after YDB. Ratios ~1 mean non-change. Shannon diversity index (H) and species richness (S) are based on raw data of all pollen and fern taxa, indicating lower richness and diversity after YDB. Vegetation turnover index is computed using the difference between contiguous samples scores of DCA Axis 1 of all pollen samples; values = 0 means no changes on vegetation, whereas values > 0 indicate a species turnover. At the YDB the vegetation turnover index increase from 0 to 0,4 supporting the vegetation change. The climatic inference based on selected taxa marked on black, indicate the dominance of open landscape during the whole section under wet/cold-temperate conditions before the YDB and the increase in seasonality of precipitation and warmer conditions after the YDB.

**Table S13. Summary of regional vegetation assemblages and environmental/climatic interpretation.**

**REFERENCES**

1 Firestone, R. B. *et al.* Evidence for an extraterrestrial impact 12,900 years ago that contributed to the megafaunal extinctions and the Younger Dryas cooling. *Proc Nat Acad Sci* **104**, 16016-16021 (2007).

2 Baker, D., Miranda, P. & Gibbs, K. in *Montana evidence for extra-terrestrial impact event that caused ice-age mammal die-off in Montana evidence for extra-terrestrial impact event that caused ice-age mammal die-off in AGU Spring Meeting Abstracts, abstract P41A-05.*

3 Fayek, M., Anovitz, L. M., Allard, L. F. & Hull, S. Framboidal iron oxide: Chondrite-like material from the black mat, Murray Springs, Arizona. *E&PSL* **319**, 251-258 (2012).

4 Ge, T., Courty, M. & Guichard, F. in *Field-Analytical approach of land-sea records for elucidating the Younger Dryas Boundary syndrome in AGU Fall Meeting Abstracts, abstract PP31D-1390.* (AGU).

5 Surovell, T. A. *et al.* An independent evaluation of the Younger Dryas extraterrestrial impact hypothesis. *Proc Nat Acad Sci* **106**, 18155-18158 (2009).

6 Laub, R. Observations from the Hiscock site (New York) bearing on a possible late-Pleistocene extraterrestrial impact event. *Curr Res Pleistoc* **27**, 168-171 (2010).

7 Mahaney, W. C. Evidence from the northwestern Venezuelan Andes for extraterrestrial impact: The black mat enigma. *Geomorphology* **116**, 48-57 (2010).

8 Mahaney, W. C., Krinsley, D. & Kalm, V. Evidence for a cosmogenic origin of fired glaciofluvial beds in the northwestern Andes: Correlation with experimentally heated quartz and feldspar. *Sediment Geol* **231**, 31-40 (2010).

9 Haynes, C. V. *et al.* The Murray Springs Clovis site, Pleistocene extinction, and the question of extraterrestrial impact. *Proc Nat Acad Sci* **107**, 4010-4015 (2010).

10 Pinter, N. *et al.* The Younger Dryas impact hypothesis: A requiem. *Earth Sci Rev* **106**, 247-264 (2011).

11 Wu, Y., Sharma, M., LeCompte, M. A., Demitroff, M. N. & Landis, J. D. Origin and provenance of spherules and magnetic grains at the Younger Dryas boundary. *Proc Nat Acad Sci* **110**, E3557-3566 (2013).

12 Pigati, J. S. *et al.* Accumulation of impact markers in desert wetlands and implications for the Younger Dryas impact hypothesis. *Proc Nat Acad Sci* **109**, 7208-7212 (2012).

13 Wright, F. W. & Hodge, P. W. Studies of particles for extraterrestrial origin: 4. Microscopic spherules from recent volcanic eruptions. *J Geophys Res* **70**, 3889-3898 (1965).

14 LeCompte, M. A. *et al.* Independent evaluation of conflicting microspherule results from different investigations of the Younger Dryas impact hypothesis. *Proc Nat Acad Sci* **109**, E2960-2969 (2012).

15 Holliday, V., Surovell, T. & Johnson, E. A blind test of the Younger Dryas impact hypothesis. *PloS One* **11**, e0155470 (2016).

16 Andronikov, A. V. *et al.* Implications from chemical, structural and mineralogical studies of magnetic microspherules from around the lower Younger Dryas boundary (New Mexico, USA). *Geogr Ann A* **98**, 39-59 (2016).

17 Luebert, F. & Pliscoff, P. *Sinopsis bioclimática y vegetacional de Chile*. 316 (Editorial Universitaria, 2006).

18 Schmithusen, J. Die raumliche Ordnung der chilenischen Vegetation. *Bonner Geogr Abh* **17** (1956).

19 Moreno, P. I., Jacobson, G. L., Lowell, T. V. & Denton, G. H. Interhemispheric climate links revealed by late-glacial cooling episode in southern Chile. *Nature* **409**, 804-808 (2001).

20 Di Castri, F. & Hajek, E. *Bioclimatología de Chile*. (Vicerrectora Acadmica, Universidad Catlica de Chile, 1976).

21 Denton, G. H. & Seno, D. Geomorphology, stratigraphy, and radiocarbon chronology of Llanquihue Drift in the area of the southern Lake , and Isla Grande de Chiloé, Chile. . *Geogr Ann A* **81**, 167-229 (1999).

22 Heusser, C. J., Heusser, L. E. & Lowell, T. V. Paleoecology of the southern Chilean Lake District-Isla Grande de Chiloé during middle-late Llanquihue glaciation and deglaciation. *Geogr Ann A* **81**, 231-284 (1999).

23 Villagrán, C. Un modelo de la historia de la vegetación de la Cordillera de La Costa de Chile central-sur: la hipótesis glacial de Darwin. *Rev Chil Hist Nat* **74**, 793-803 (2001).

24 Lamy, F. *et al.* Antarctic timing of surface water changes off Chile and Patagonian ice sheet response. *Science* **304**, 1959-1962 (2004).

25 Rojas, M., Moreno, P., Kageyama, M., Crucifix, M. & Hewitt, C. The Southern Westerlies during the last glacial maximum in PMIP2 simulations. *Clim Dyn* **32**, 525-548 (2009).

26 Moreno, P. I., Lowell, T. V., Jacobson Jr, G. L. & Denton, G. H. Abrupt vegetation and climate changes during the last glacial maximumand last termination in the chilean lake district: a case study from canal de la puntilla (41° s). *J Quat Sci* **81**, 285-311 (1999).

27 Villagrán, C. Late quaternary vegetation of southern Isla Grande de Chiloé, Chile. . *Quat Res* **29**, 294-306 (1988).

28 Villagrán, C. Expansion of Magellanic moorland during the late Pleistocene: palynological evidence from northern Isla de Chiloe, Chile. . *Quat Res* **30**, 304-314 (1988).

29 Labarca, R., Recabarren, O. P., Canales-Brellenthin, P. & Pino, M. The gomphotheres (proboscidea: Gomphotheriidae) from Pilauco site: Scavenging evidence in the Late Pleistocene of the Chilean Patagonia. *Quat Int* **352**, 75-84 (2014).

30 Moreno, P. I., Abarzúa, A. M. & Villagrán, C. Deglacial and postglacial climate history in east-central Isla Grande de Chiloé, southern Chile (43°S). *Quat Res* **62**, 49-59 (2004).

31 Hajdas, I., Bonani, G., Moreno, P. I. & Ariztegui, D. Precise radiocarbon dating of Late-Glacial cooling in mid-latitude South America. *Quat Res* **59**, 70-78 (2003).

32 Pesce, O. & Moreno, P. Vegetation, fire and climate change in central-east Isla Grande de Chiloé (43 S) since the Last Glacial Maximum, northwestern Patagonia. *Quat Sci Rev* **90**, 143-157 (2014).

33 Jara, I. A. & Moreno, P. I. Climatic and disturbance influences on the temperate rainforests of northwestern Patagonia (40° S) since∼ 14,500 cal yr BP. *Quat Sci Rev* **90**, 217-228 (2014).

34 Moreno, P. I. *et al.* Radiocarbon chronology of the last glacial maximum and its termination in northwestern Patagonia. *Quat Sci Rev* **122**, 233-249 (2015).

35 Moreno, P. I. & Videla, J. Centennial and millennial-scale hydroclimate changes in northwestern Patagonia since 16,000 yr BP. *Quat Sci Rev* **149**, 326-337 (2016).

36 Abarzúa, A. M. & Moreno, P. I. Changing fire regimes in the temperate rainforest region of southern Chile over the last 16,000 yr. *Quat Res* **69**, 62-71 (2008).

37 Schilling, D. H. & Hollin, J. T. in *Numerical reconstructions of valley glaciers and small ice caps in The Last Great Ice Sheets.* Vol. 207 220 (Wiley, 1981).

38 Villagrán, C. Análisis palinológico de los cambios vegetacionales durante el Tardiglacial y Postglacial en Chiloé, Chile. *Rev Chil Hist Nat* **58**, 57-69 (1985).

39 Moreno, P. I. & León, A. L. Abrupt vegetation changes during the last glacial to Holocene transition in mid‐latitude South America. *J Quat Sci* **18**, 787-800 (2003).

40 Ariztegui, D., Bianchi, M. M., Masaferro, J., Lafargue, E. & Niessen, F. Interhemispheric synchrony of Late-glacial climatic instability as recorded in proglacial Lake Mascardi, Argentina. . *J Quat Sci* **12**, 333-338 (1997).

41 Ashworth, A. C. & Markgraf, V. Climate of the Chilean channels between 000 to 10,000 yr BP based on fossil beetle and pollen analyses *Rev Chil Hist Nat* **62**, 61-74 (1989).

42 Moreno, P. I. Climate, Fire, and Vegetation between About 13,000 and 9200 14 C yr B.P. in the Chilean Lake District. *Quat Res* **54**, 81-89 (2000).

43 Heusser, C. J. Late-glacial-Holocene climate of the Lake District of Chile. *Quat Res* **22**, 77-90 (1984).

44 Batist, M. D., Fagel, N., Loutre, M. & Chapron, E. A 17,900-year multi-proxy lacustrine record of Lago Puyehue (Chilean Lake District): introduction. . *J Paleolimnol* **39**, 151-161 (2008).

45 Vargas-Ramirez, L., Roche, E., Gerrienne, P. & Hooghiemstra, H. A pollen-based record of late glacial-Holocene climatic variability in the southern Lake District, Chile. *J Paleolimnol* **39**, 197-217 (2008).

46 Pino, M., Chávez-Hoffmeister, M., Navarro-Harris, X. & Labarca, R. The late Pleistocene Pilauco site, Osorno, south-central Chile. *Quat Int* **299**, 3-12 (2013).

47 Pino, M., Martel-Cea, A., Vega, R., Fritte, D. & Soto-Bollmann, K. in *Geología y geomorfología del sitio Pilauco in El Sitio Pilauco. Osorno, Patagonia Noroccidental de Chile.* 12-46 (Universidad Austral de Chile Valdivia, Chile, 2016).

48 Tello, F., Elgueta, M., Torres, F. & Pino, M. Abarzúa, A. M., Fossil beetles from Pilauco, south-central Chile: An Upper Pleistocene paleoenvironmental reconstruction. *Quat Int* **449**, 58-66 (2017).

49 Bryan, A. L. & Gruhn, R. Some difficulties in modeling the original peopling of the Americas. *Quat Int* **109**, 175-179 (2003).

50 Gnecco, C. & Aceituno, J. Poblamiento temprano y espacios antropogénicos en el norte de Suramérica. *Complutum* **15**, 151-164 (2004).

51 Mazz, J. M. L. Early human occupation of Uruguay: Radiocarbon database and archaeological implications. *Quat Int* **301**, 94-103 (2013).

52 Nami, H. G. Archaelogy, Paleoindian Research and Lithic Technology in the Middle Negro River, Central Uruguay. *Archaeological Discovery* **1**, 1-22 (2013).

53 Boëda, E. *et al.* The late-Pleistocene industries of Piauí, Brazil: new data. *Paleoamerican Odyssey*, 445-465 (2013).

54 Dillehay, T. D. Entangled knowledge: Old trends and new thoughts in first South American studies. *Paleoamerican odyssey*, 377-395 (2013).

55 Aceituno, F. J., Loaiza, N., Delgado-Burbano, M. E. & Barrientos, G. The initial human settlement of Northwest South America during the Pleistocene/Holocene transition: Synthesis and perspectives. *Quat Int* **301**, 23-33 (2013).

56 Schulte, P. *et al.* The Chicxulub asteroid impact and mass extinction at the Cretaceous-Paleogene boundary. *Science* **327**, 1214-1218 (2010).

57 Schultz, Zarate, Hames, Camilion & King. A 3.3-Ma impact in argentina and possible consequences. *Science* **282**, 2061-2063 (1998).

58 Vizcaíno, S. F., Fariña, R. A., Zárate, M. A., Bargo, M. S. & Schultz, P. Palaeoecological implications of the mid-Pliocene faunal turnover in the Pampean Region (Argentina). *Palaeogeogr Palaeoclimatol Palaeoecol* **213**, 101-113 (2004).
